# The dorsal chaetotaxy of *Trogolaphysa* (Collembola, Paronellidae), with descriptions of two new species from caves in Belize

**DOI:** 10.3897/zookeys.323.4950

**Published:** 2013-08-13

**Authors:** Felipe N. Soto-Adames, Steven J. Taylor

**Affiliations:** 1 Illinois Natural History Survey, University of Illinois, 1816 S Oak St, Champaign IL 61820 USA

**Keywords:** Puerto Rico, *Dicranocentruga*, phylogeny, cave-adaptive characters

## Abstract

Species diagnosis in *Trogolaphysa* has been based, until now, almost exclusively on number of eyes and shape of claws and mucro. Chaetotaxy, a character system important to diagnose species in other genera of scaled Entomobryoidea, has been described only for a few *Trogolaphysa* species. Here the complete dorsal chaetotaxy of six species of *Trogolaphysa* is described using the AMS and Szeptycki’s systems for head and body, respectively. A morphology-based parsimony analysis was performed to evaluate whether chaetotaxic characters overcome the influence of putatively cave adaptive convergent characters to resolve species level relationships, and to evaluate the evolution of the dorsal macrochaetae of the head. Phylogenetic analysis using only putative cave-adaptive characters support clades of unrelated taxa, but the addition of chaetotaxy overcomes the influence of convergent characters. A phylogeny based on all characters supports a trend towards reduced head macrochaetae number. Head macrochaetae are lost beginning with A3 and followed, in order, by S5, S3 and M3. In addition, a checklist of New World *Trogolaphysa* is provided and two new species, *Trogolaphysa giordanoae* **sp. n.** and *Trogolaphysa jacobyi* **sp. n.**, are described on the basis of material collected in six caves in southern Belize.

## Introduction

The collembolan fauna of Belize is among the least known of any Central American country. The Catalogue of Neotropical Collembola (Mari Mutt and Bellinger 1990) and subsequent updates (Mari Mutt and Bellinger 1996, Mari Mutt et al. 2009) list one species, the troglomorphic *Trogolaphysa belizeana* Palacios-Vargas & Thibaud, 1997, for Belize. A recent biospeleological expedition to the Toledo District of Belize yielded several new springtail species, including two new species in the genus *Trogolaphysa*.

What is understood about the evolution of morphological adaptations to cave habitats in entomobryoid springtails is derived from northern temperate members of the genera *Pseudosinella* and *Sinella* (Christiansen 1961, Gama 1984). The evolution of troglobiont species in tropical *Trogolaphysa* (Palacios Vargas et al. 1985[1986]) and *Troglopedetes* (Deharveng 1987, Deharveng and Gers 1993), could provide important independent information to test hypotheses about the direction of character evolution in Entomobryoidea. The characters utilized in the descriptions of most of the 33 species of *Trogolaphysa* reported from the New World (Mari Mutt and Bellinger 1990, Mari Mutt et al. 2009; Table 1) are limited to claw complex and mucronal shape (e.g., Palacios-Vargas et al. 1985[1986]), two character systems prone to convergent evolution in cave habitats (Christiansen 1961, Christiansen and Culver 1987). Chaetotaxy is known for few species (Gruia 1987, Mari Mutt 1987[1988], Thibaud and Najt 1988[1989]), and is limited to the number of macrochaetae. While convergence itself is of interest in understanding evolution in caves (Derkarabetian et al. 2010, Hedin and Thomas 2010), distinguishing convergent characters adaptive for subterranean life from characters that better reflect phylogenetic history has proven to be important in a variety of groups of animals (e.g., Wiens et al. 2003).

**Table 1.** Check-list of the species of *Trogolaphysa* *sensu* Thibaud and Najt (1988[1989]) of the New World, with distribution by country (given as ISO 3166–1 alpha-3 code).

**Species**	**Distribution**
*Trogolaphysa aelleni* Yoshii, 1988	BRA
*Trogolaphysa belizeana* Palacios-Vargas & Thibaud, 1997	BLZ
*Trogolaphysa berlandi* (Denis, 1925)	ARG, GUF
*Trogolaphysa bessoni* Thibaud & Najt, 1989	ECU
*Trogolaphysa caripensis* (Gruia, 1987)	VEN
*Trogolaphysa carpenteri* (Denis, 1925)	CRI, GUF, MEX, VEN
*Trogolaphysa cotopaxiana* Thibaud & Najt, 1989	ECU
*Trogolaphysa distinguenda* (Denis, 1931)	CRI
*Trogolaphysa ecuatorica* (Palacios-Vargas, Ojeda & Christiansen, 1986)	ECU
*Trogolaphysa geminata* (Mari Mutt, 1988)	PRI
*Trogolaphysa giordanoae* Soto-Adames & Taylor, sp. n.	BLZ
*Trogolaphysa guacharo* Yoshii, 1988	CRI, VEN
*Trogolaphysa haitica* (Palacios-Vargas, Ojeda & Christiansen, 1986)	HTI
*Trogolaphysa hauseri* Yoshii, 1988	BRA
*Trogolaphysa hirtipes* (Handschin, 1924)	ARG, BRA, VEN
*Trogolaphysa hondurasensis* (Palacios-Vargas, Ojeda & Christiansen, 1986)	HND
*Trogolaphysa jamaicana* (Palacios-Vargas, Ojeda & Christiansen, 1986)	JAM
*Trogolaphysa jataca* (Wray, 1953)	JAM, PRI
*Trogolaphysa jacobyi* Soto-Adames & Taylor, sp. n.	BLZ
*Trogolaphysa luquillensis* (Mari Mutt, 1988)	PRI
*Trogolaphysa marimutti* (Palacios-Vargas, Ojeda & Christiansen, 1986)	MEX
*Trogolaphysa maya* Mills, 1938	CUB, DOM, MEX
*Trogolaphysa millsi* Arlé, 1939	BRA
*Trogolaphysa nacionalica* (Palacios-Vargas, Ojeda & Christiansen, 1986)	MEX
*Trogolaphysa oztotlica* (Ojeda & Palacios-Vargas, 1984)	MEX
*Trogolaphysa relicta* (Palacios-Vargas, Ojeda & Christiansen, 1986)	MEX
*Trogolaphysa riopedrensis* (Mari Mutt, 1988)	PRI
*Trogolaphysa separata* (Denis, 1933)	CRI
*Trogolaphysa strinatii* Yoshii, 1988	MEX
*Trogolaphysa subterranea* (Mari Mutt, 1988)	PRI
*Trogolaphysa tijucana* (Arlé & Guimarāes, 1979)	BRA
*Trogolaphysa toroi* (Palacios-Vargas, Ojeda & Christiansen, 1986)	MEX
*Trogolaphysa variabilis* (Palacios-Vargas, Ojeda & Christiansen, 1986)	MEX
*Trogolaphysa xtolokensis* (Palacios-Vargas, Ojeda & Christiansen, 1986)	MEX
*Trogolaphysa yoshiia* (Palacios-Vargas, Ojeda & Christiansen, 1986)	MEX

The relationships between the genera *Paronella*, *Troglopedetes*, *Trogolaphysa*, and *Dicranocentruga* has been a source of confusion. Thibaud and Najt (1988[1989]) evaluated morphological characters of these genera and provided clear diagnoses for all of them: *Paronella* was retained for species with 1+1 rows of external spines on the manubrium; *Troglopedetes* was restricted to species with a single subdivision of the fourth antennal segment; *Trogolaphysa* was circumscribed to include *Paronella*-like species with Ant. 4 not subdivided, manubrium without spines and a short mucro (in relation to dens) with 3-5 teeth; whereas *Dicranocentruga* was placed as a junior synonym of *Trogolaphysa*. Thibaud and Najt (1988[1989]) did not consider the presence of EOS (extra ocular structure) as a diagnostic character. Mitra (1992, 1993, 2002) argued that species without manubrial spines but sharing the presence of an EOS and 8+8 (or apparently 6+6) eyes should be placed in the genus *Dicranocentruga*, whereas species with fewer than 6+6 eyes and without EOS should be allocated to *Trogolaphysa* or *Troglopedetes*. Mitra (1993) suggested that further observations of the chaetotaxy would furnish characters to support this separation, but until now, the complete dorsal chaetotaxy of these taxa remained undescribed.

Here we present complete descriptions of the dorsal chaetotaxy of the head and trunk for the two new species of *Trogolaphysa* and for *Trogolaphysa belizeana*, andcompare their chaetotaxy to that of *Trogolaphysa jataca* (Wray, 1953), *Trogolaphysa geminata* (Mari Mutt, 1987[1988]) and *Trogolaphysa riopedrensis* (Mari Mutt, 1987[1988]), three surface species from Puerto Rico. Finally, we present a morphology-based phylogenetic analysis to assess the value of chaetotaxy in elucidating species relationships in this genus, and to evaluate the evolution of some elements of the dorsal chaetotaxy of the head.

## Materials and methods

Springtails were collected with aspirators and preserved in 70% ethanol. Samples were associated with substrate characterizations and field-collected measurements of temperature, light intensity and humidity.

Selected specimens were cleared in Nesbitt’s solution, mounted in Mark André II (Mari Mutt 1979) on glass slides, and examined under a compound microscope with phase contrast. The extra-ocular structure (EOS) was examined under polarized light. Drawings were made using a drawing tube, with final illustrations completed using Adobe Illustrator CS5, version 15.0.2.

Abbreviations used for structures are: antennae (Ant.), thorax (Th.), abdomen (Abd.), extra ocular structure (EOS). Abbreviations used for names are: Avelardo Canti (AC), Gabriel Chaco (GaC), Germano Coe (GeC), William R. Elliott (WRE), Geoff B. Hoese (GBH), JoAnn Jacoby (JJ), Jean K. Krejca (JKK), Bruno K. Kuppinger (BKK), C. Marcela Ospina (CMO), Rosalio Sho (RS), Christy M. Slay (CMS), Michael E. Slay (MES), Felipe N. Soto-Adames (FSA), and Steven J. Taylor (SJT).

To protect vulnerable sites, latitude and longitude are not provided for the Belize material. These locations are controlled by, and may be requested from, the Institute of Archaeology, Belmopan, Belize (see Acknowledgements). Holotypes and paratypes of the new species are deposited in the Illinois Natural History Survey Insect Collection (INHS).

Here we describe only elements of the chaetotaxy that are modified into microchaetae, macrochaetae or sensilla (i.e., idiochaetotaxy, Szeptycki 1979). We follow the nomenclature of Szeptycki (1979) for the body and the AMS system (Jordana and Baquero 2005, Soto-Adames 2008, 2010) for the head. Mitra (1993) proposed a system for the chaetotaxy of the head in Paronellidae, but it has not been widely embraced, whereas the AMS system has been applied to entomobryoids since the 1970’s (Szeptycki 1973, Mari Mutt 1979) and allows evaluation of homologies between families of Entomobryoidea.

The idiochaetotaxy of *Trogolaphysa* is reduced, and in naming body setae we assume it represents the remnant of primary chaetotaxy. The setae closest to the mesothoraxic pseudopore (Figs 12, 32, 53) are identified as m1 and m2, even though they occupy positions that in entomobryoids with more abundant idiochaetotaxy might be assigned to setae m2i and m2e, respectively. The nomenclature of setae on the fourth abdominal segment follows Szeptycki’s system (Soto-Adames 2010): setae in columns A and B are named sequentially from posterior to anterior, irrespective of their relative insertion. Columns A and B have a maximum of four setae, and when all are present they are always setae 3-6 (e.g., A3, A4, A5 and A6). In the species described below, setae A3, A6, B3 and B6 are always present, and it is assumed that setae A4 and B4 are always suppressed before A5 and B5.

For the labial chaetotaxy, upper case letters represent macro- or mesosetae and lower case represent microsetae, an underscore in the formula identifies ciliate setae. The eye patch of a generalized springtail comprises a group of 5 anterior and 3 posterior simple eyes, we refer to the space between these two groups of eyes as the ‘eye patch well’ to distinguish it from the inter-ocular space, which is the gap between the eye patches on either side of the head.

The formula of the dorsal macrochaetae of head and trunk is based on Gisin’s (1967) model, but we consider all macrochaetae associated with the bothriotricha on abdominal segments 2-4, instead of only those found between the bothriotrichal complexes. The number of macrochaetae on the head is presented as two digits; the first digit refers to macrochaetae anterior to the head sulcus (series A, M and S), the second to the posterior macrochaetae (series Ps, Pa and Pm). The macrochaetae on abdominal segment 4 are represented by three digits separated by plus (+) symbol, where the first, second and third numbers refer to the inner (series A and B), medial (assumed, in Szeptycki’s system, to be series C) and outer macrochaetae (series T, D, E, F and Fe). The last number in the macrochaeta formula may be represented by a range because the number of outer macrochaetae may be variable, as some macrochaetae external to series F appear to be added as individuals grow older. The formula is based on the relative size of the sockets and includes all macrochaetae, irrespective of whether they are large (i.e., short, thick and blunt) or small (long, slender and acuminate).

Phylogenetic trees were estimated using parsimony as implemented in PAUP 4.0* (Swofford 2002).

The habitat parameters substrate temperature, air temperature, light, and relative humidity were measured with hand held meters. Differences in abiotic parameters between habitats occupied by the two new species were tested using a Wilcoxon rank sum test in R 2.15.2 (R Developmental Core Team 2012), with continuity correction.

## Results

### Genus Trogolaphysa

Mills, 1938 sensu Thibaud and Najt (1988[1989])

http://species-id.net/wiki/Trogolaphysa

#### Diagnosis.

Paronellidae with finely denticulate scales covering dorsum of head and body, and ventral face of furcula; Ant. 4 sometimes annulated, never subdivided in two; labial seta L2 normal, not reduced; eyes 0-8; EOS present; Abd. 2-4 with 2, 3, 3 bothriothricha; manubrium without spines, dens with 1-2 rows of spines; mucro short, with 3-5 more or less evenly spaced teeth.

#### Remarks.

As currently circumscribed (Thibaud and Najt 1988[1989]), the absence of a subdivision on Ant. 4 in *Trogolpahysa* is the only character that distinguishes this genus from *Troglopedetes*.

It is not known if the type species of the genus, *Trogolaphysa maya* Mills, 1938, has EOS, but the presence of this structure in all species discussed below, including the two troglomorphic forms, suggests it is likely also present in that species.

### Trogolaphysa giordanoae

Soto-Adames & Taylor sp. n.

http://zoobank.org/3C37791A-056D-496F-87E2-58D72A355B4B

http://species-id.net/wiki/Trogolaphysa_giordanoae

Figs 1 –21 ; Figs 22 –23 (habitat)

#### Type locality.

BELIZE: Toledo District: 29 km WNW of Punta Gorda, Blue Creek Cave, Hokeb Ha entrance, 11.IV.2011, SJT, MES, JJ, CMS, GBH & RS, coll.

**Type material**:Holotype, female on microscope slide preparation, INHS Collection Number 579,406; Paratypes: BELIZE: Toledo District: 29 km WNW of Punta Gorda, Blue Creek Cave, Hokeb Ha entrance, 11.IV.2011, (3 in alcohol), SJT, MES, JJ, CMS, GBH & RS, coll.; 37 km WNW of Punta Gorda, cave near Pueblo Creek Cave, 16.IV.2011, (4 in alcohol-one headless), MES, JKK, CMS, GBH & GeC, coll.; 28 km NNW of Punta Gorda, Tiger Cave, 9.IV.2012, (1 on slide, 33 in alcohol), SJT, MES, JJ, CMS, GBH, BKK & GaC, coll.; 28 km NNW of Punta Gorda, Bat Cave, 10.IV.2011, (2 on slides, 29 in alcohol—some in poor condition, one headless), SJT, MES, JJ, CMS & GBH, coll.; 31 km WNW of Punta Gorda, Okebal Ha, 14.IV.2011, (3 on slides, 16 in alcohol), SJT, MES, JJ, CMS, GBH, BKK & RS, coll.

#### Diagnosis.

*Trogolaphysa giordanoae* sp. n. is unique among species with 6–8 eyes in having 5 dorsal head macrochaetae, 3 metathoracic macrochaetae and 4 inner macrochaetae on Abd. 4. Among species with known dorsal chaetotaxy, the new species is most similar to *Trogolaphysa riopedrensis*, but the two species are easily distinguished by the combination of characters given above and by the presence of a relatively shorter mucro in the new species (Table 2). Additional diagnostic characters distinguishing the new species from all other New World *Trogolaphysa* with 6–8 eyes and capitate/spatulate tenent hair are presented in Table 2. Among the species described before the introduction of chaetotaxy, the new species is most similar to *Trogolaphysa distinguenda* (Denis, 1931), but the two species can be separated by the presence of a relatively long mucro with 5 teeth in *distinguenda*, and a 4-toothed short mucro in *Trogolaphysa giordanoae* sp. n. *Trogolaphysa belizeana* is the only other New World *Trogolaphysa* with 3 metathoracic macrochaetae. However, *Trogolaphysa belizeana* is a troglobiont (*sensu* Sket 2008, Culver and Pipan 2009)—blind, with long antennae and modified ungues.

**Table 2.** Diagnostic table for species of *Trogolaphysa* with 6–8 eyes and capitate or spatulate tenent hair.

**Species**	**Mucronal teeth**	**Mucro length/ Width dens apex**	**Inner ungual teeth**	**Dorsal head macrochaetae**	**Th. 2 Macrochaetae**	**Th. 3 Macrochaetae**	**Abd. 4 Inner large macrochaetae**
*Trogolaphysa giordanoae* sp. n.	4	1.8	4	5	7	3	4
*Trogolaphysa riopedrensis*	4	2.9	4	7	7	0	4
*Trogolaphysa geminata*	4	2.2	4	6	7	0	3
*Trogolaphysa jataca*	4	2.9	4	7	7	0	3
*Trogolaphysa carpenteri* †	4	3.5	3	2	0	0	0
*Trogolaphysa relicta*	4	2.7	3	0	0	0	0
*Trogolaphysa subterranea*	4	2.7	3	3	7	0	3
*Trogolaphysa cotopaxiana*	5	3.6	4	2	3	0	3
*Trogolaphysa distinguenda*	5	3.3	4	?	?	?	?

† Most characters based on Yoshii (1988).

#### Description.

**Size.** Body length up to 2.1 mm.

**Figure 1.**
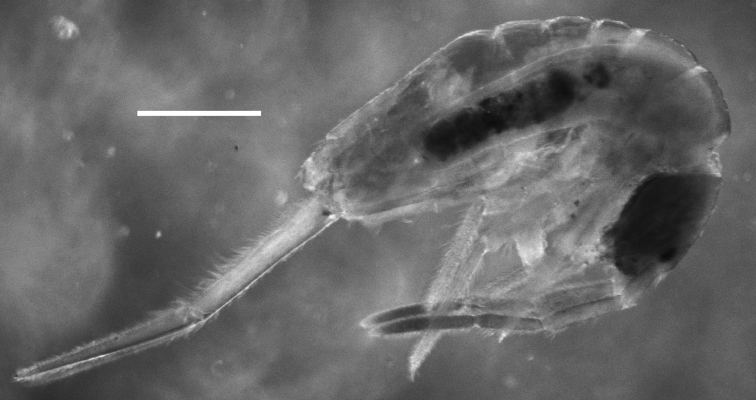
*Trogolaphysa giordanoae* sp. n. habitus, scale=0.5 mm.

**Color.** Pattern, if any, obscured by green dye present in the alcohol in which specimens were preserved (Fig. 1).

**Scale distribution.** Scales dark brown, present on Ant. 1-2 and base of Ant. 3, more abundant on dorsal face than on ventral face of segment. Scales absent from ventral tube, legs and dorsal face of manubrium.

**Figures 2–10.**
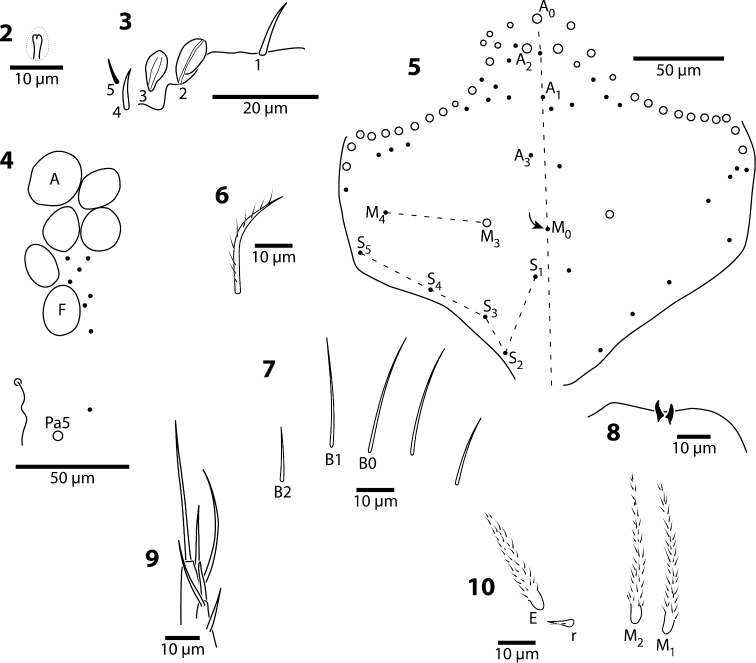
*Trogolaphysa giordanoae* sp. n., circles are macrochaetae, filled circles are ciliate microchaetae**2** Antennal segment 4, subapical sensillum **3** Antennal segment 3, sense organ **4** Eyepatch and associated setae, **5** Head dorsal chaetotaxy, line represents dorsal sulcus **6** Prelabral seta **7** Labral row B setae **8** Distal margin of labrum **9** Labial papilla E **10** Posterior setae of labial triangle.

**Figures 11–13.**
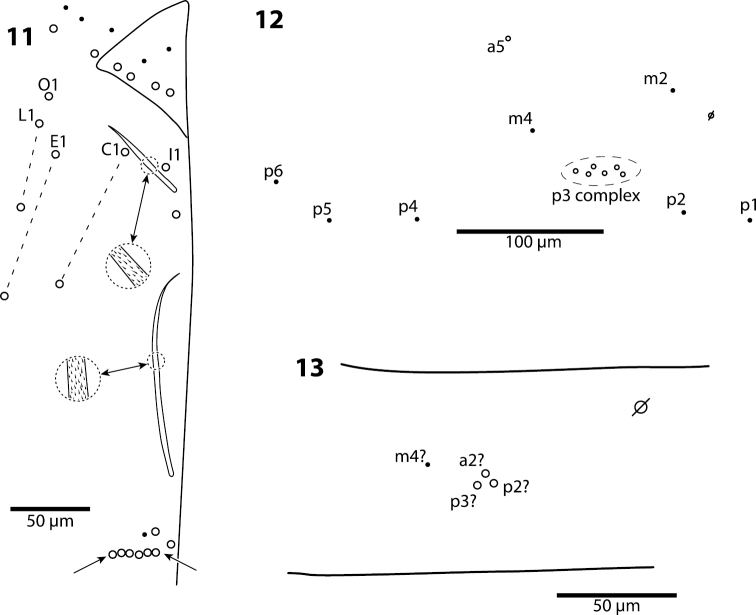
*Trogolaphysa giordanoae* sp. n. **11** Postlabium, circles are ciliate setae, filled circles are smooth setae, arrows point at ventral cervical setae **12** Mesothorax, dorsal chaetotaxy, circles are macrochaetae, filled circles are microchaetae **13** Metathorax, dorsal chaetotaxy, circles are macrochaetae, filled circles are microchaetae, seta a6 present but not shown.

**Head.** Antenna/cephalic diagonal ratio 2.0–2.5 (Fig. 1). Apical bulb of Ant. 4 absent; subapical sensillum capitate (Fig. 2), fully contained in circular depression; guard sensillum absent. Sense organ of Ant. 3 (Fig. 3) with sensilla 1 and 4 acuminate, thin-walled and translucent; sensillum 5 acuminate, dark (light dense), shorter than 1 and 4; sensilla 2–3 wide, leaf-like, resting in shallow grooves. Eyes 6+6 (Fig. 4), chaetotaxy of eyepatch well with 4, sometimes 6 ciliate setae, and 1 seta posterior to eye F. Head dorsally with 5 macrochaetae (A0, A2, M3, Pa5 and Pm3 — Figs 4–5). Series M with 2 setae (M3–4); series S with 5 setae (S1–5); seta M0 seen only in one individual; S0 absent. Prelabral setae serrate (Fig. 6). Labral setae smooth: setae in rows A and C subequal; seta B2 distinctly shorter than setae B0 and B1 (Fig. 7). Distal margin of labrum with 1+1 medial hooks, papillae absent (Fig. 8). Apical and subapical setae of maxillary palp smooth; sublobular plate with 2 seta-like appendages. Lateral process of labial papilla E weakly bent dorsally, barely reaching apex of papilla (Fig. 9). Labial triangle setae as M1M2rEL1–2A1–5 (Fig. 10); r short, stout and sparsely ciliate; L1 inserted close to E and distant from L2 when compared to other entomobryoids (Fig. 11). Postlabium covered by setae and scales, all postlabial setae ciliate, modified setae absent. Columns ICELO with 42221 setae (Fig. 11): column I with posterior seta detached from main group and much longer than anterior setae. Ventral cervical setae usually 8+8.

**Figures 14–16.**
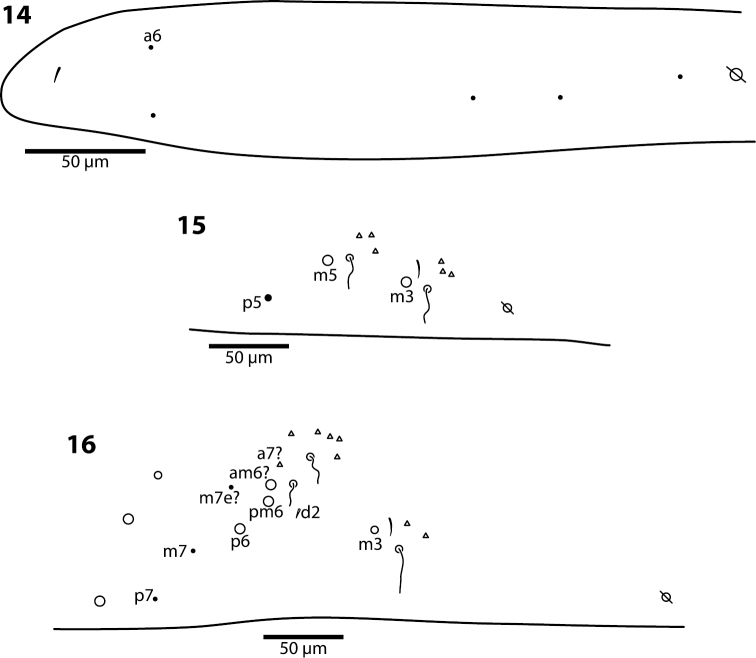
*Trogolaphysa giordanoae* sp. n. Dorsal chaetotaxy of abdominal segments 1–3, triangles are fan-shaped setae, circles are macrochaetae, filled are circles ciliate microchaeta**14** First abdominal segment **15** Second abdominal segment **16** Third abdominal segment.

**Figures 17–21.**
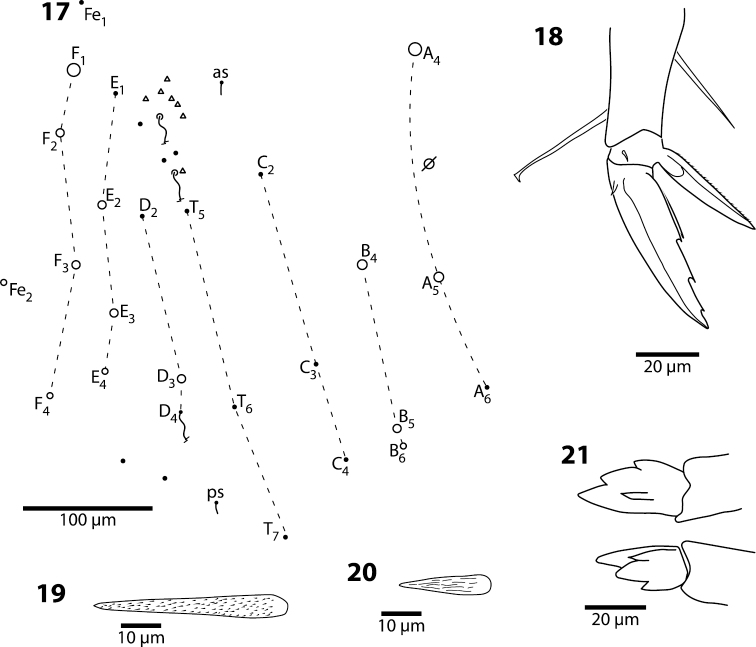
*Trogolaphysa giordanoae* sp. n. **17** Fourth abdominal segment dorsal chaetotaxy, diameter of circle is approximately proportional to size of macrochaeta **18** Metathoracic claw complex **19** Dens basal spine, outer row **20** Dens basal spine, inner row **21** Mucro.

**Body.** Mesothoracic hood not developed. Complete dorsal macrochaetae as 32/73/0245+0+9. Mesothorax with 1 anterior (a5) and 6 posterior (p3 complex) macrochaetae arranged as is typical for genus (Fig. 12); microchaetae m2, m4, p1, p2, p4, p5 and p6 present. Inner chaetotaxy of metathorax with 3 macro- and 1 microchaetae (Fig. 13). First abdominal segment with 1 anterior (a6) and 4 posterior setae arranged in a single row (Fig. 14). Second abdominal segment (Fig. 15) inner bothriotrix with 3 fan-shaped setae, one microsensillum and macrochaeta m3; outer bothriotrix with 3 fan-shaped setae and macrochaeta m5; setae a6, m6 and p5 present. Third abdominal segment (Fig. 16) inner bothriotrix complex with 2 fan-shaped setae, 1 sensillum, and macrochaeta m3; external bothriotricha with 7 fan-shaped setae, and macrochaetae am6, pm6 and p6; sensillum d2 present, inserted near pm6. Fourth abdominal segment with 5 inner and 9 outer macrochaetae (Fig. 17): large inner macrochaetae A4, A5, B4, and B5 present; B6 a small macrochaeta; large outer macrochaetae D3, E2, E3, F1, F2, and F3 present; macrochaetae E4, F4 and one other seta probably homologous to Fe4, small. Anterior and medial bothriotricha with 7 and 3 fan-shaped supplementary setae, respectively. Posterior bothriotrix, corresponding to D4, without associated supplementary setae. Posterior setae 19–21+19–21. Intersegmental membrane between Abd. 4–5 with 4–10 lenticular organs (as in *Trogolaphysa riopedrensis*, Fig. 60).

**Legs.** Trochanteral organ with up to 36 setae. Metathoracic claw complex as in Fig. 18. Tenent hair weakly spatulate. Smooth posterior setae on metathoracic legs 0.76× as long as unguiculus. Unguis with 4 inner teeth: 1 basal tooth sometimes appearing slightly larger than other, both paired teeth ending near middle of inner edge; proximal unpaired tooth as large as basal paired teeth, ending on distal half of inner edge; distal unpaired tooth smallest of all inner teeth and ending on distal fourth of inner edge. Outer tooth ending on basal quarter of outer ungual edge. Unguiculus lanceolate, with outer margin serrate.

**Ventral tube**. Anterior face with 3+3 or 4+4 distal macrochaetae; lateral and posterior setae not seen clearly.

**Furcula**. Dens with 2 rows of ciliate spines: inner row with 35–42 spines; outer row with 25–28 spines. Basal outer spines longest (Figs 19–20). Mucro with 4 short, stout teeth (Fig. 21), ratio mucro length/width of dens tip 1.2–1.8×; basal outer tooth reaches to at least half length of basal inner tooth.

#### Etymology.

This species is dedicated to Rosanna Giordano, the senior author’s wife, for her years of support and contributions to science.

#### Distribution.

The species is known only fromBelize

#### Habitat.

**Figure 22.**
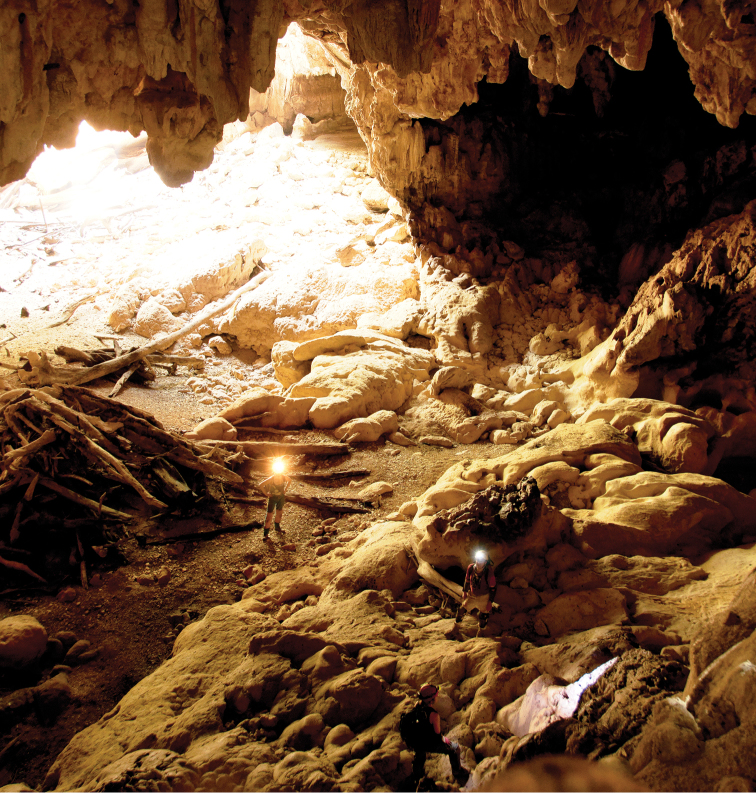
*Trogolaphysa giordanoae* sp. n. paratype habitat Okebal Ha entrance/twilight zone. Specimens were collected from a small pile of fruit bat guano near the researchers in the foreground, below a bat roost site. Sample site was much darker than it appears in this enhanced image. Photo courtesy of MES.

**Figure 23.**
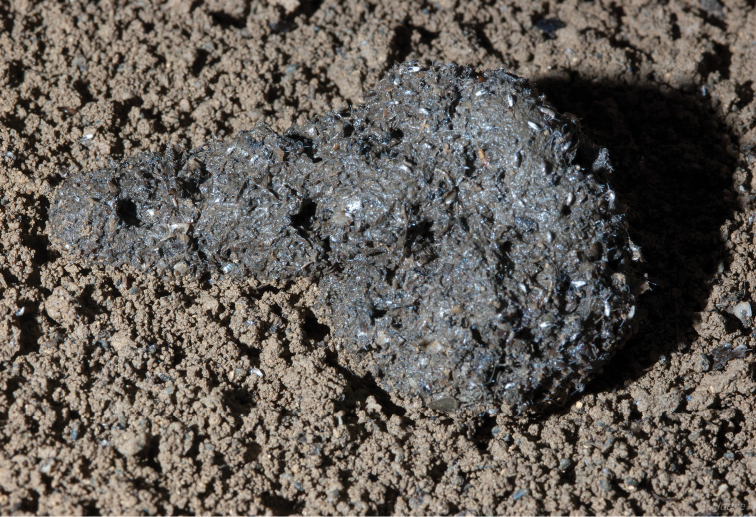
*Trogolaphysa giordanoae* sp. n. on old feces in Tiger Cave. Photo courtesy of GBH.

*Trogolaphysa giordanoae* sp. n. is a guanophile, recorded from entrance, twilight (Fig. 22) and dark zones of caves (6.7, 53.3 & 40.0 % of 15 collections, respectively), often in association with fruit bat or other guano (Fig. 23) (noted for 40% of 15 collections). It was commonly found on the floor of caves (76.9% of 13 collections where position was noted), but also on cave walls (23.1% of 13 collections where position was noted).

### Trogolaphysa jacobyi

Soto-Adames & Taylor sp. n.

http://zoobank.org/5F865EE9-B5E0-4844-8482-902F8E9EA2B2

http://species-id.net/wiki/Trogolaphysa_jacobyi

Figs 24 –43 ; Fig. 44 (habitat)

#### Type locality.

BELIZE: Toledo District: 32 km WNW of Punta Gorda, Yok Balum Cave, 13.IV.2012, SJT, MES, JJ, CMS, GBH & AC, coll.

#### Type material.

Holotype, female on microscope slide preparation, INHS collection number 579,407; BELIZE: Toledo District: 32 km WNW of Punta Gorda, Yok Balum Cave, 13.IV.2012, SJT, MES, JJ, CMS, GBH & AC, coll.; Paratypes: BELZE: Toledo District: 32 km WNW of Punta Gorda, Yok Balum Cave, 13.IV.2012, (2 adults & 1 juvenile on slides, 3 adults or subadults & 3 juveniles in alcohol), SJT, MES, JJ, CMS, GBH & AC, coll.; 37 km WNW of Punta Gorda, cave near Pueblo Creek Cave, 16.IV.2011, (1 adult on slide—without legs), MES, JKK, CMS, GBH & GeC, coll.

#### Diagnosis.

*Trogolaphysa jacobyi* sp. n. is the only member of the genus that is blind, has 3-toothed mucro and unguis, and has a single macrochaeta on the metathorax. *Trogolaphysa belizeana* is the only other New World *Trogolaphysa* lacking eyes and having a 3-toothed mucro, but it differs from *Trogolaphysa jacobyi* sp. n. in having 3 metathoracic macrochaetae (1 in *Trogolaphysa jacobyi* sp. n.), in the arrangement and identity of inner macrochaetae on Abd. 4 (cf. Figs 38, 49 see discussion below), in having few postlabial scales (absent in *Trogolaphysa jacobyi* sp. n.) and setae (many in *Trogolaphysa jacobyi* sp. n., cf. Figs 30, 46), in the presence of sensillum d2 on Abd. 3 (absent in *Trogolaphysa jacobyi* sp. n.), in the absence of unpaired ungual teeth (1 tooth in *Trogolaphysa jacobyi* sp. n.) and in having a typical lanceolate unguiculus (basally swollen in *Trogolaphysa jacobyi* sp. n.). Table 3 provides a list of characters that distinguish *Trogolaphysa jacobyi* sp. n. from all other New World *Trogolaphysa* lacking eyes and having paired basal ungual teeth inserted near the basal fourth of the inner edge.

**Table 3.** Diagnostic table for blind species of *Trogolaphysa* with basal paired ungual teeth originating on basal fourth of inner edge of claw.

**Species**	**Mucronal teeth**	**Inner ungual teeth**	**Unguiculus shape**	**Mesothorax macrochaetae**	**Metathorax macrochaetae**	**4^th^ Abdominal segment large inner macrochaetae**
*Trogolaphysa jacobyi* sp. n.	3	3	basally swollen	4	1	A5, B4, B5
*Trogolaphysa belizeana*	3	2	lanceolate	4	3	A4, A5, B5
*Trogolaphysa haitica*	4	2	lanceolate	0	0	0
*Trogolaphysa ecuatoriana*	5	2	basally swollen	0	0	0
*Trogolaphysa bessoni*	5	2	basally swollen	3	0	apparently<br/> A5, B4, B5

#### Description.

**Size.** Body length up to 2.0 mm.

**Figure 24.**
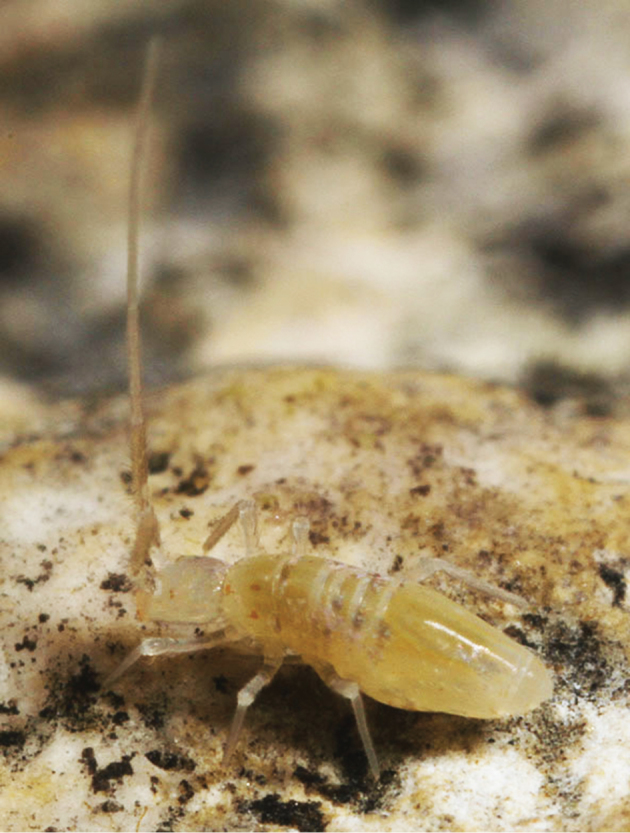
*Trogolaphysa jacobyi* sp. n. habitus, photographed in Yok Balum Cave.

**Color.** Living specimens yellowish, with pigment only on a small eyepatch and mesothorax (Fig. 24). Specimens in alcohol white, without trace of pigment.

**Scale distribution.** Scales transparent, present on Ant. 1–2. Scales absent from postlabial region of head, ventral tube, legs and dorsal face of manubrium.

**Figure 25.**
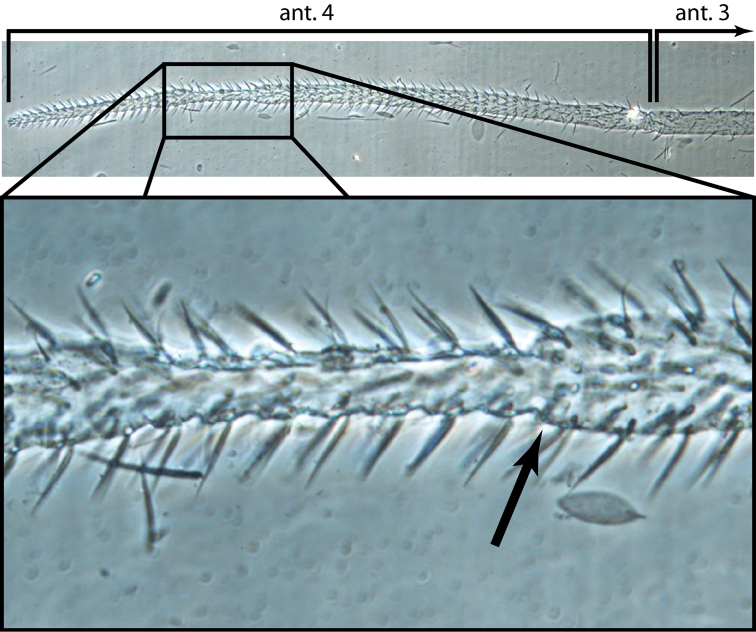
*Trogolaphysa jacobyi* sp. n. Fourth antennal segment showing constriction and incomplete suture (arrow).

**Figures 26–29.**
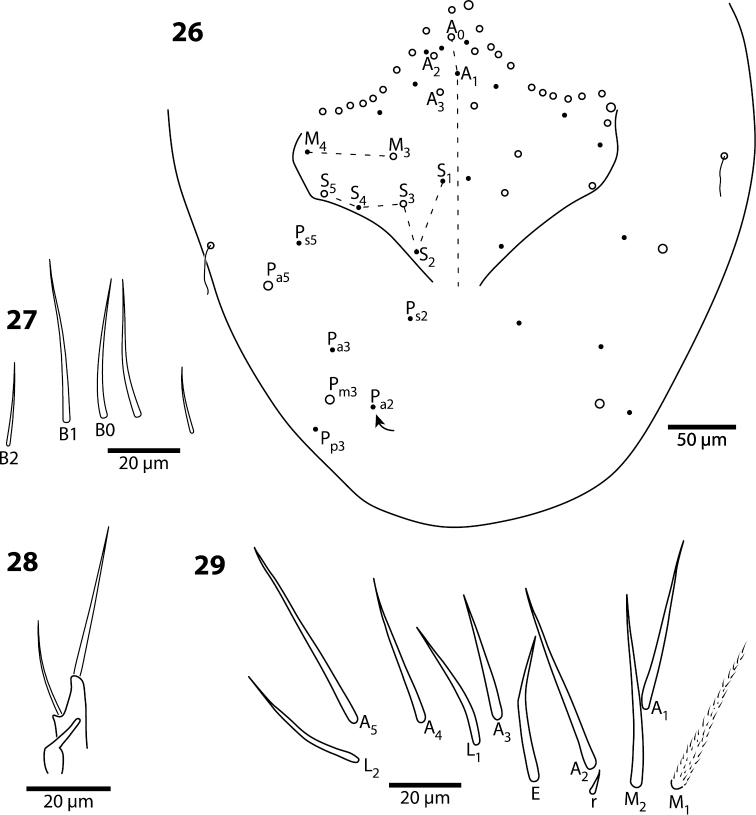
*Trogolaphysa jacobyi* sp. n. **26** Head dorsal chaetotaxy **27** Labral setae on row B **28** Lateral process of labial papilla E **29** Labial triangle.

**Figures 30–32.**
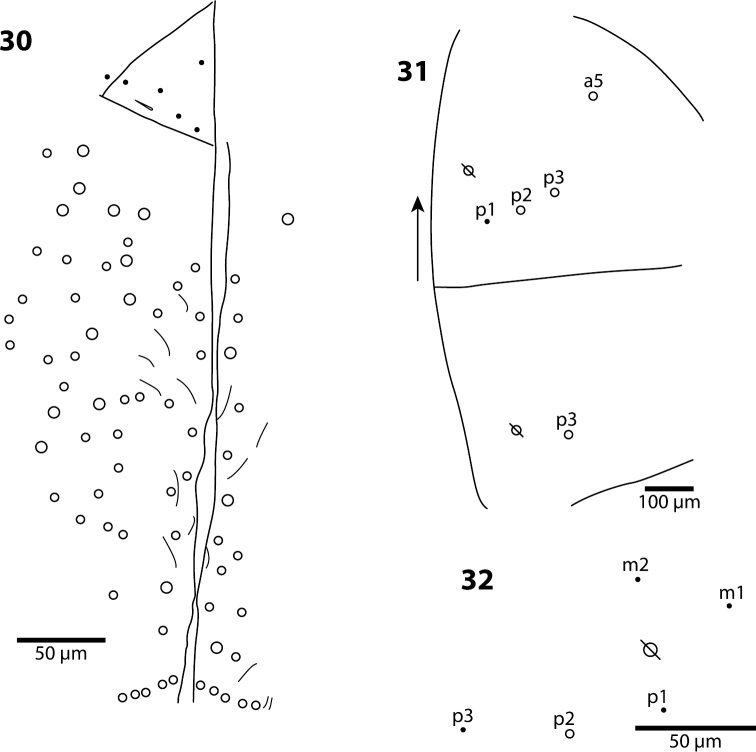
*Trogolaphysa jacobyi* sp. n. **30** Labial triangle and postlabium, open and filled circles represent ciliate and smooth setae, respectively **31** Thorax macrochaetae **32** Mesothorax, detail of inner chaetotaxy on a different individual.

**Head.** Antenna/cephalic diagonal ratio up to 5.8 (Fig. 24). Fourth antennomere with incomplete but clear constriction near middle, with many shallow whorls of setae (Fig. 25); apical bulb absent; subapical sensillum not seen. Sense organ of Ant. 3 with sensilla 1 and 4 short, acuminate, thin-walled and translucent; sensillum 5 acuminate, dark and shorter than 1 and 4; sensilla 2–3 broad, leaf-like, resting in shallow grooves. Eyes not seen on slide-mounted specimens, but 1–2 pigment patches visible in life (Fig. 24). Head dorsally with 8 macrochaetae (A0, A2, A3, M3, S3, S5, Pa5 and Pm3 — Fig. 26). Seta M4 displaced laterally towards cephalic sulcus. Series S with setae S1–5; S0 absent, macrochaeta S3 displaced anteriorly, away from cephalic sulcus (cf. Figs 5, 26). Prelabral and all labral setae smooth: setae within row A and C subequal; seta B2 shorter than B0 and B1 (Fig. 27). Distal margin of labrum smooth, papillae absent. Apical and subapical setae of maxillary palp smooth; sublobular plate without seta-like appendages. Lateral process of labial papilla E weakly bent dorsally and not nearly reaching apex of papilla (Fig. 28). Labial triangle setae as M1M2rEL1–2A1–5 (Fig. 29), seta M1 ciliate, all others smooth; r short; A2 close to r, L1 close to E and distant from L2. Postlabium without scales, polychaetotic, uniformly covered with many large and small, weakly ciliate or smooth setae (Fig. 30); modified setae absent. Columns ICELO ill defined due to polychaetosis. Ventral cervical setae usually 6+6.

**Figures 33–37.**
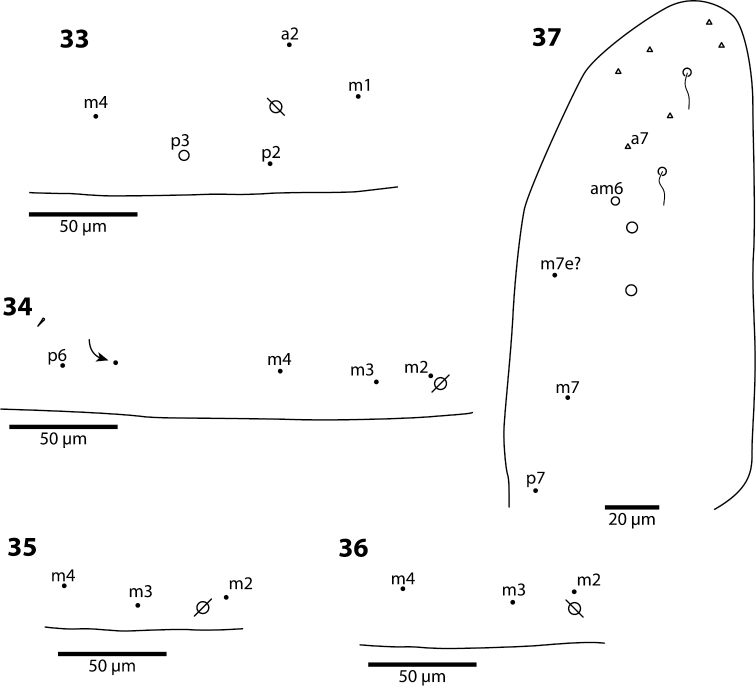
*Trogolaphysa jacobyi* sp. n., open and filled circles represent macro- and microchaetae, respectively, triangles represent fan-shaped microchaetae. **33** Metathorax, detail of inner chaetotaxy, seta a6 present but not shown **34** First abdominal segment, chaetotaxy, arrow points at seta seen in a single individual **35–36** First abdominal segment, alternative insertions of seta m2 **37** Third abdominal segment, setae associate with lateral bothriotricha.

**Figures 38–43.**
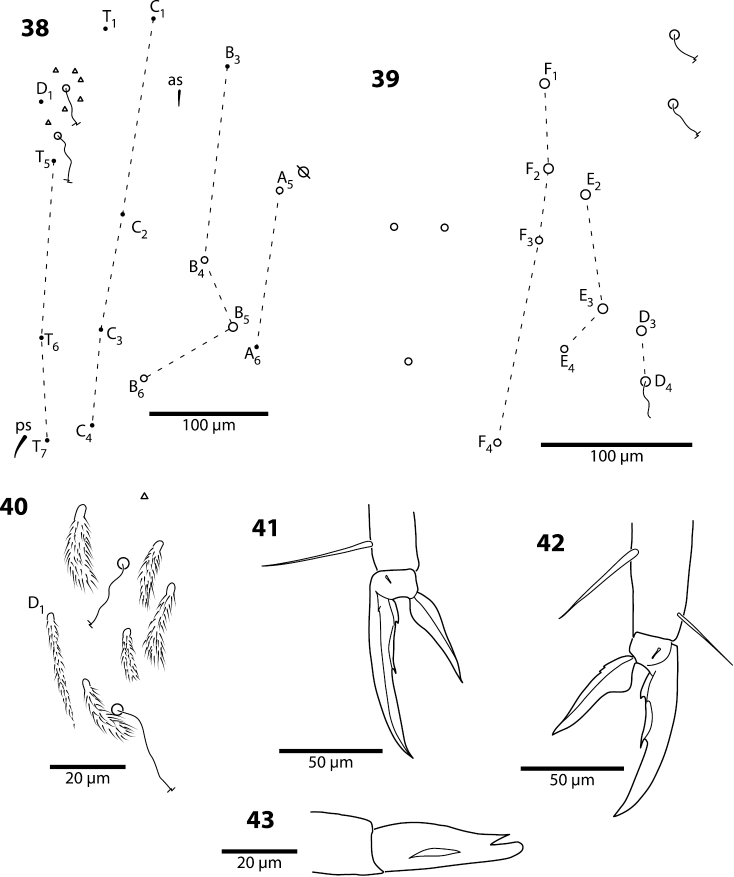
*Trogolaphysa jacobyi* sp. n., symbols as in previous plate. **38** Fourth abdominal segment, inner chaetotaxy **39** Fourth abdominal segment, outer macrochaetae **40** Fourth abdominal segment anterior bothriotrichal complex **41** Prothoracic claw complex **42** Metathoracic claw complex **43** Mucro.

**Body.** Mesothoracic hood not developed. Complete dorsal macrochaetae as 62/41/0244+0+9-11. Mesothorax with 1 anterior (a5) and usually 3 posterior (p1–3) macrochaetae forming an arch (Fig. 31); some individuals with only mesothoraxic macrochaeta p2 (Fig. 32); microchaetae m1, m2, m4, p4 and p5 present. Metathorax with 1 macro- and 5 microchaetae (Fig. 33). First abdominal segment seta a6 absent; 4 posterior setae arranged in a single row (Fig. 34–36). Inner bothriotrix complex of Abd. 2 with 3 fan-shaped setae, one microsensillum and macrochaeta m3; outer bothriotrix with 3 fan-shaped setae and macrochaeta m5; setae a6, m6 and p5 present. Inner bothriotrix complex of Abd. 3 with 3 fan-shaped setae, one sensillum and macrochaetae m3; external bothriotrichal complex (Fig. 37) with 6–7 fan-shaped setae, macrochaetae am6, pm6 and p6; sensillum d2 absent. Fourth abdominal segment with 4 inner (Fig. 38) and 9–11 outer (Fig. 39) macrochaetae: inner macrochaetae A5, B4, and B5 large, B6 small; B5 displaced towards A6 instead of B6; (Fig. 38). Outer macrochaetae D3, E2, E3, F1, and F2 large; small outer macrochaetae E4, F3, F4 and 3 others probably belonging to series Fe present. Abd. 4 anterior and medial bothriotricha with 4 and 2 fan-shaped supplementary setae, respectively (Fig. 39). Posterior bothriotrix corresponds to D4, without associated supplementary setae. Posterior setae 6–7+6–7. Intersegmental membrane between Abd. 4–5 with 4–7 lenticular organs.

**Legs.** Trochanteral organ with up to 25 setae. Claw complex as in Figs 41–42. Tenent hair acuminate, longer on L1 than L3. Smooth posterior setae on metathoraxic legs as long as unguiculus. Unguis with 3 inner teeth: basal teeth small, subequal and ending on basal fourth of inner edge; unpaired tooth distinctly larger than basal teeth, ending near middle of inner ungual edge. Outer tooth absent on all claws; lateral teeth present only on pro- and mesothoracic legs, and ending on basal quarter of outer edge of unguis (Fig. 41). Unguiculus basally swollen, with basal fifth of outer margin weakly serrate.

#### Ventral tube.

Anterior face with 2+2 distal macrochaetae; lateral and posterior setae not seen.

**Furcula.** Dens with 2 rows of finely ciliate spines, number of spines per row unclear on all specimens examined, but inner row with at least 36 spines. Mucro elongate and slender, with 3 teeth, basal inner tooth absent (Fig. 43): ratio mucro length/width of dens tip 2.3–2.8 (mode=2.4).

#### Distribution.

The species is known only from caves in southern Belize

#### Remarks.

*Trogolaphysa jacobyi* sp. n. is a troglobiont (*sensu* Sket 2008, Culver and Pipan 2009). Living specimens seem to have eye pigment (Fig. 24), but we were unable to identify corneas on specimens examined. The only structure resembling a cornea corresponds to the EOS.

It could be argued that the constriction of the fourth antennomere places this species in *Troglopedetes*. However, the presence of a well-developed ciliate labial seta L2, the incomplete nature of the constriction on Ant. 4, and the similarity with *Trogolaphysa belizeana* (presumably with complete, unconstricted Ant. 4, and therefore an uncontested *Trogolaphysa*) suggest that *Trogolaphysa jacobyi* sp. n. should be retained in *Trogolaphysa*. Additionally, the fact that all other *Troglopedetes* species are restricted to the Old World have prompted us to retain the new species in *Trogolaphysa*.

#### Etymology.

This species is dedicated to JoAnn Jacoby, the junior author’s wife, in gratitude for her enthusiasm and assistance in the planning and execution of field-work in the caves of Belize and in many earlier excursions.

#### Habitat.

**Figure 44.**
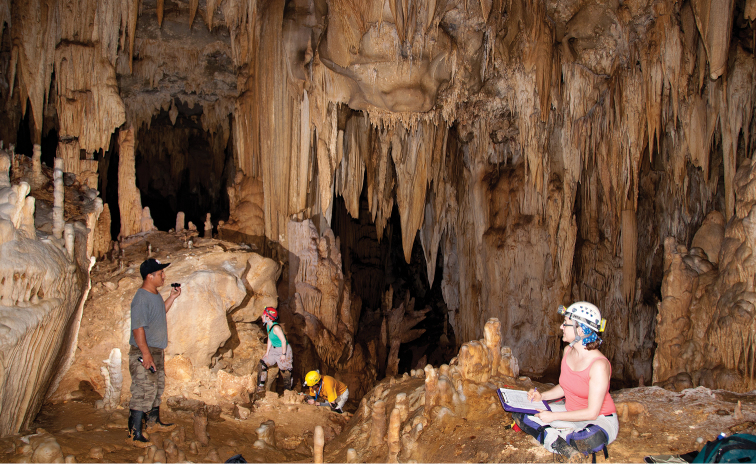
Type locality for *Trogolaphysa jacobyi* sp. n. in the dark zone of Yok Balum. Photo courtesy of MES.

This species is a troglobiont, and all 5 collections (11 individuals) were taken in the dark zone (0 lux) on the floor (Fig. 44), often (80% of collections) in wet conditions associated with flowstone or calcite and drip pools, sometimes with scattered cricket droppings.

### Trogolaphysa belizeana

Palacios-Vargas & Thibaud, 1997

http://species-id.net/wiki/Trogolaphysa_belizeana

Figs 45 –52

#### Material examined.

Two paratypes; Belize: Cayo District, Actun Chapal cave, 7 km SE of Benque Viejo del Carmen, 10.XII.1992, W.R. Elliott.

Additions to the original description.

**Figures 45–49.**
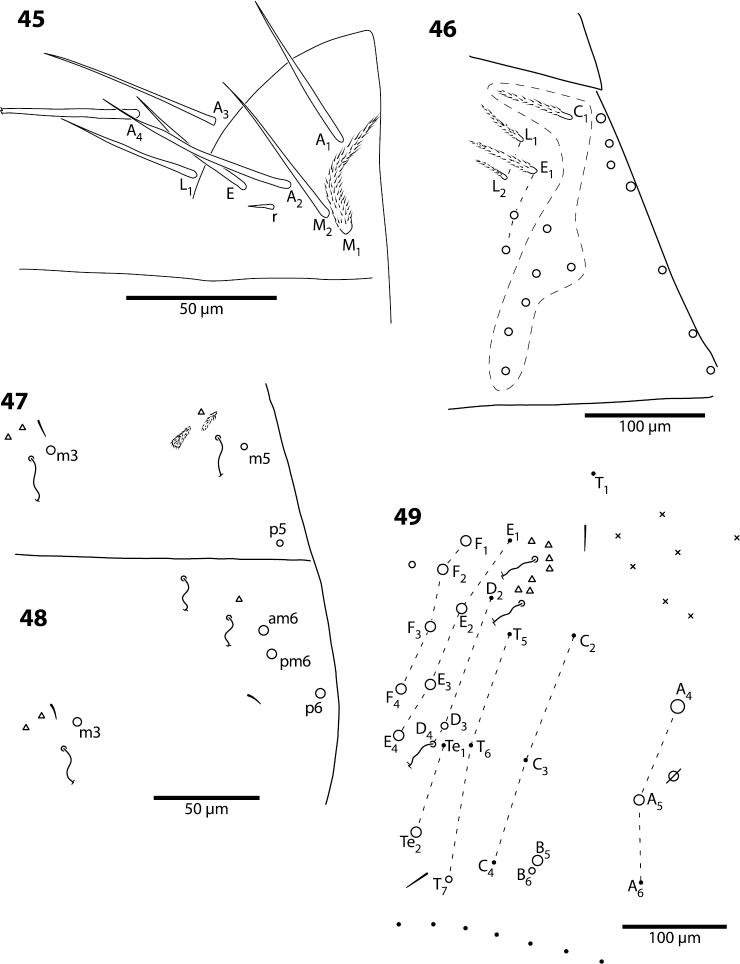
*Trogolaphysa belizeana* **45** Labial triangle **46** Postlabial chaetotaxy **47** Chaetotaxy of second abdominal segment **48** Chaetotaxy of third abdominal segment **49** Complete chaetotaxy of fourth abdominal segment, x represent sensilla-like setae.

**Head.** Dorsal chaetotaxy of the head identical to that of *Trogolaphysa jacobyi* sp. n., with macrochaetae A0, A2, A3, M3, S3, S5, Pa5 and Pm3. Labral margin smooth. Sublobular plate of outer maxillary lobe without setae-like appendages. Labial papilla E with lateral appendage reaching tip of papilla; 5 proximal smooth labial setae present, seta z (Soto-Adames 2010) longest. Labial triangle formula as M1M2rEL1L2A1-5 (Fig. 45): M1 ciliate, shorter but thicker than M2; r short, stout, apically acuminate; A2 close to r. Postlabium with few scales; columns ICEL with 7732 setae (Fig. 46); seta L2 shortest; ventral cervical setae 6+6.

**Body.** Dorsal macrochaeta formula as 62/3–43/0343+0+11. Mesothorax with macrochaetae p2, p3 and a5, and microchaetae m4 and p5 clearly visible; setae p1, m2 and p6 obscured. Metathorax with 3 macro- and 1 microchaetae arranged as in *Trogolaphysa giordanoae* sp. n. (Fig. 13). Abd. 1 with at least three inner microchaetae, apparently without a6, but lateral field of segment not clearly visible. Abd. 2 chaetotaxy normal (Fig. 47): with bothriotricha m2 and a5, sensillum as, macrochaetae m3 and m5, setae a6, m6 and p5, and fan-shaped supplementary setae around bothriotrichal complexes. Abd. 3 (Fig. 48) with insertion of bothriotricha m2, a5 and m5, macrochaetae m3, a7, pm6 and p6, and sensillum d2 normally placed. Chaetotaxy of Abd. 4 as in Fig. 49: inner macrochaetae A4, A5, B5 and B6 present, B6 smallest; outer macrochaetae T7, D3, E2, E3, E4, F1, F2, F3, F4, one member of series Fe and one posterior setae of unclear homology present; relative position of bothriotricha normal; microchaeta B4 absent, microchaeta Te1 present. Posterior setae 7+7. Intersegmental membrane between Abd. 4–5 with at least 4 lenticular organs, actual number of organs unclear due to folding of membrane.

**Figures 50–52.**
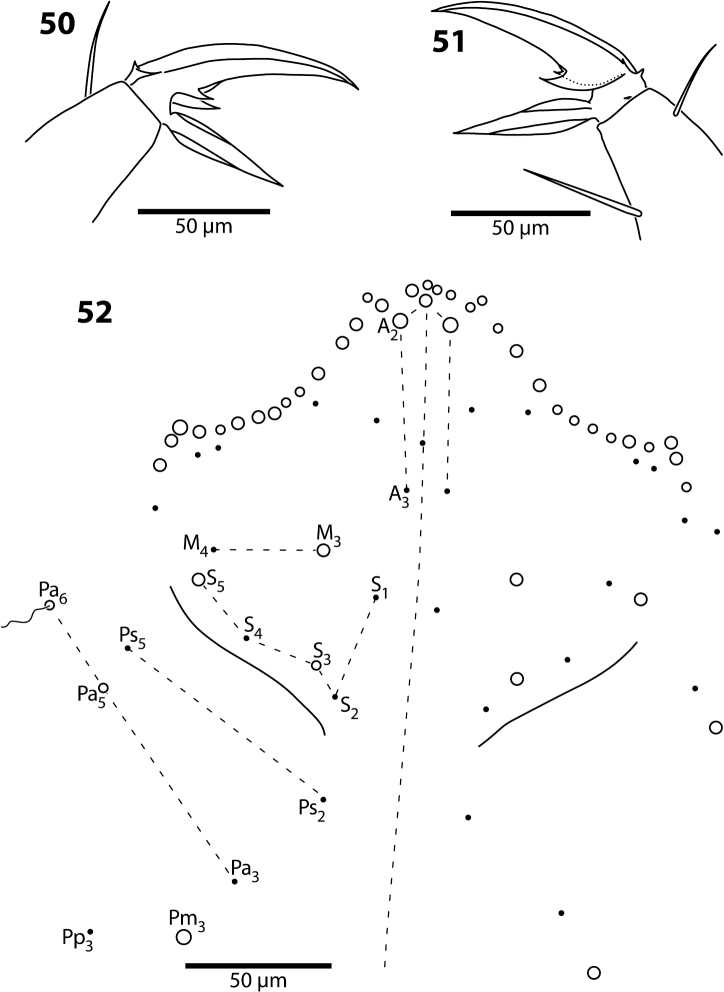
*Trogolaphysa belizeana* (**50**, **51**) and *Trogolaphysa jataca* (**52**) **50** Prothoracic claw **51** Metathoracic claw **52** Dorsal chaetotaxy of head.

**Legs.** Claw complex of pro- and metathoracic legs as in Figs 50–51. Tenent hair acuminate. Outer and lateral unguis teeth small, inconspicuous; inner paired teeth with one tooth slightly, but clearly larger, unpaired teeth absent. Unguiculus lanceolate.

**Ventral tube.** With 2+2 distal macrochaetae on anterior face.

#### Remarks.

The paratypes examined differ from the original description of the species in having labial seta L2 smooth instead of ciliate, in having only 2 posterior mesothoraxic macrochaetae, in the claws having lateral teeth and in the number of bothriotricha on Abd. 2 and Abd. 4.

Variation in the number of mesothoraxic macrochaetae is also seen in *Trogolaphysa jacobyi* sp. n and may be related to post-embryonic development. The chaetotaxy of Abd. 2 in fig. 12 of Palacios-Vargas and Thibaud (1997) suggests a composite of the chaetotaxy of Abd. 2 and 3, whereas the bothriotrichal complex of Abd. 4 shown in Palacios-Vargas and Thibaud (1997, fig. 13) seems based on an aberrant specimen.

### Trogolaphysa jataca

(Wray, 1953)

http://species-id.net/wiki/Trogolaphysa_jataca

Fig. 53–55

#### Material examined.

Puerto Rico: Isabela, Guajataca Commonwealth Forest, Rd. 446, 18.41263°N, 66.96887°W, top of mogote at crossroad between trails 6, 25 & 26, leaf litter, 15.V.2009, F. Soto (2 specimens); Cayey, Rd. 4471, Km 4.1, leaf litter, 18.VI.1998, F. Soto (1 specimen); Mayagüez, University of Puerto Rico, secondary forest east of Biology Building, 18.21350°N, 67.13774°W, royal palm (*Roystonea borinquena* O.F. Cook) leaf litter, III.2009, M. Ospina (3 specimens).

Additions to the original description.

**Head.** Dorsal chaetotaxy as in Fig. 52: macrochaetae A0, A2, M3, S3, S5, Pa5 and Pm3 present; 1+1 microchaetae inserted near A1. Postlabium with all setae ciliate; columns ICELO with 41232; posterior setae on column I detached from anterior group.

**Figures 53–55.**
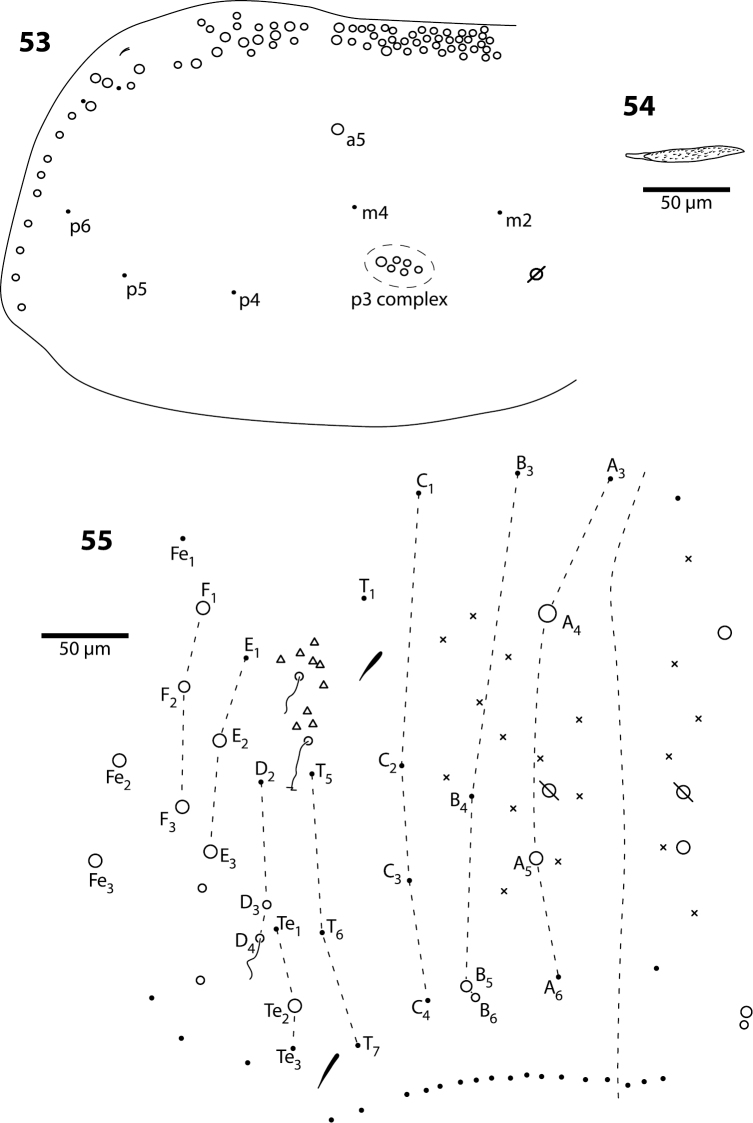
*Trogolaphysa jataca* **53** Mesothorax chaetotaxy **54** Second abdominal segment seta p5 **55** Complete chaetotaxy of fourth abdominal segment.

**Body.** Mesothorax (Fig. 53) with one anterior (a5) and six posterior macrochaeta; microchaetae m2, m4, p5 and p6 present; microchaetae p1 and p2 absent. Metathorax with 4 inner microchaetae, as in *Trogolaphysa riopedrensis* (Fig. 58). Abd. 1 with 4 posterior setae; seta a6 absent. Abd. 2 and 3 as in *Trogolaphysa giordanoae* sp. n. (Figs 15, 16); Abd. 2 seta p5 fusiform, with enlarged socket (Fig. 54). Abd. 4 as in Fig. 55: inner macrochaetae A4, A5, B5 and B6 present; macrochaetae Te2, D3, E2, E3, F1–3 present; 4 other lateral and posterior small macrochaetae present. Posterior setae 13–14+13–14. Intersegmental membrane between Abd. 4–5 with 4 lenticular organs.

**Ventral tube.** Anterior face with 3+3 distal macrochaetae; smaller individuals with 2+2 macrochaetae.

### Trogolaphysa geminata

(Mari Mutt, 1988)

http://species-id.net/wiki/Trogolaphysa_geminata

Fig. 56

#### Material examined.

Puerto Rico: Maricao, Maricao Commonwealth Forest, near observation tower on Rd. 120, 18.14444°N, 66.97962°W, leaf litter, 8.VI.1998, F. Soto (1 specimen); Mayagüez, University of Puerto Rico, secondary forest east of Biology Building, 18.21350°N, 67.13774°W, royal palm (*Roystonea borinquena*) leaf litter, III.2009, M. Ospina (3 specimens).

Additions to the original description.

**Figures 56–58.**
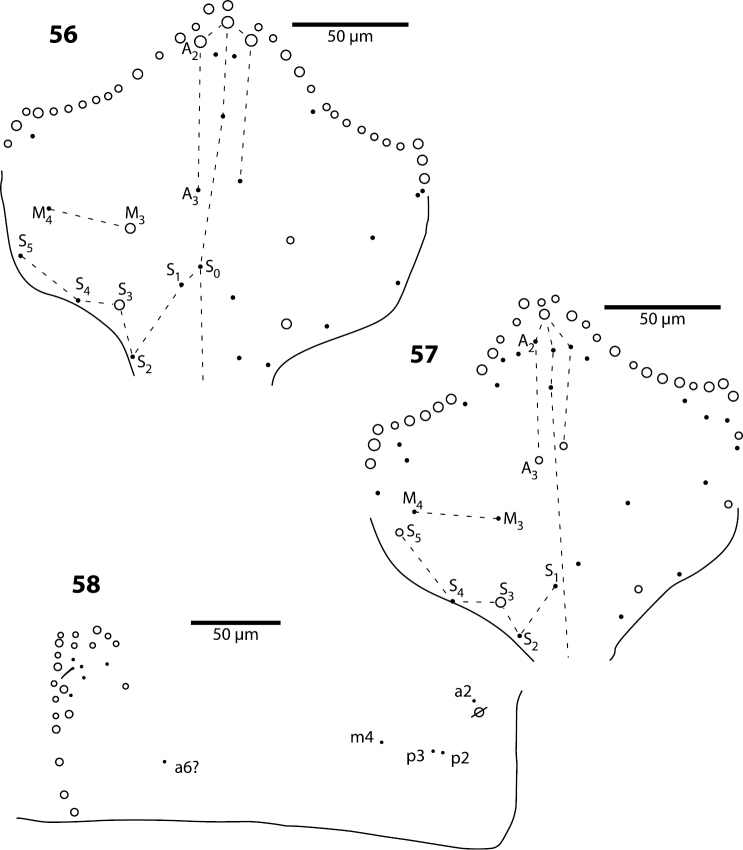
*Trogolaphysa geminata* (**56**) and *Trogolaphysa riopedrensis* (**57, 58**) **56** Head dorsal chaetotaxy **57** Head dorsal chaetotaxy **58** Metathorax chaetotaxy.

**Head.** Dorsal chaetotaxy as in Fig. 56: macrochaetae A0, A2, M3, S3, Pa5 and Pm3 present. Postlabium with all setae ciliate; columns ICELO with 41232; posterior setae on column I detached from anterior group.

**Figures 59, 60.**
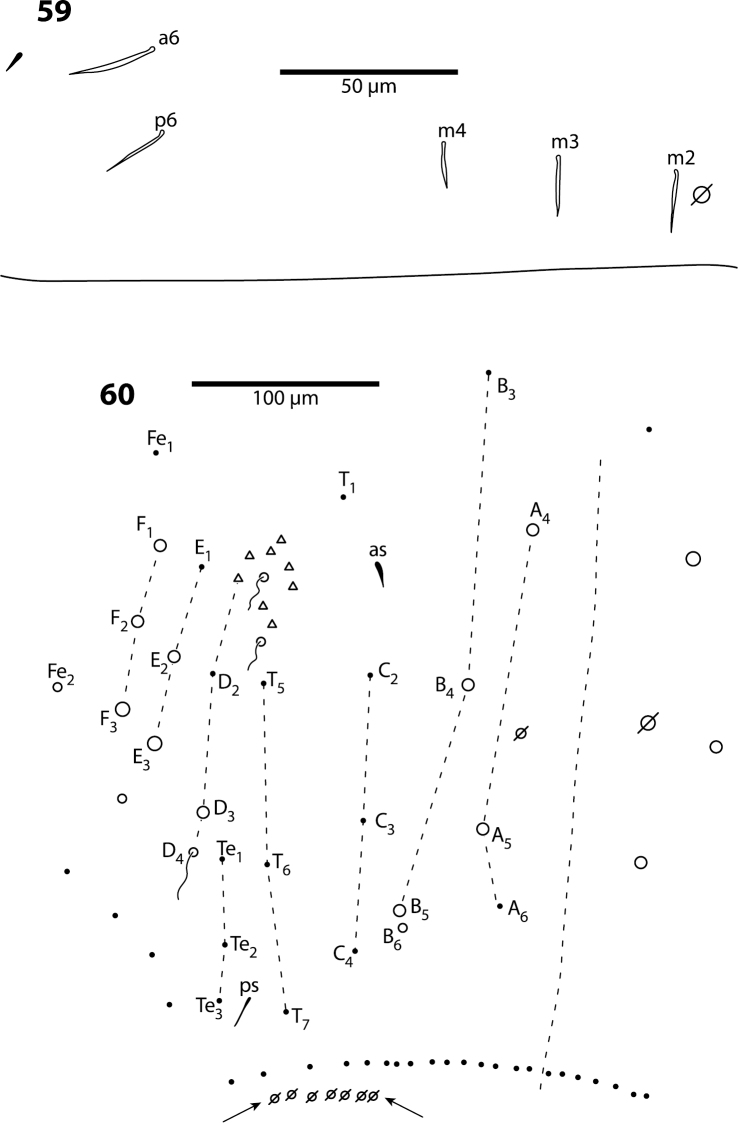
*Trogolaphysa riopedrensis* **59** Chaetotaxy of first abdominal segment **60** Complete chaetotaxy of fourth abdominal segment, arrows identify the lenticular organs.

**Body.** Mesothorax as in *Trogolaphysa jataca* (Fig. 53). Metathorax as in *Trogolaphysa riopedrensis* (Fig. 58). Abd. 1 as in *Trogolaphysa riopedrensis* (Fig. 59) with one anterior (a6) and 4 posterior setae. Abd. 2 and 3 as in *Trogolaphysa giordanoae* sp. n. (Figs 15, 16); Abd. 2 seta p5 fusiform as in *Trogolaphysa jataca*. Abd. 4 as in *Trogolaphysa jataca* (Fig. 55): inner macrochaetae A4, A5, B5 and B6 present; macrochaetae Te2, D3, E2, E3, F1, F2, F3 present; 4 other lateral and posterior small macrochaetae present. Posterior setae 13–14+13–14. Intersegmental membrane between Abd. 4–5 with 4–6 lenticular organs.

**Ventral tube.** Anterior face with 3+3 distal macrochaetae.

### Trogolaphysa riopedrensis

(Mari Mutt, 1988)

http://species-id.net/wiki/Trogolaphysa_riopedrensis

Fig. 57 –60

#### Material examined.

Puerto Rico, Aguadilla, Caimital Alto, Villa Grajales, 18.44058°N, 67.11840°W, moist mown lawn, 9.VII.1999, F. Soto (1 specimen); USA Virgin Islands, St. Thomas, 18.35348°N, 64.93520°W, wet leaf litter, patch of forest along Rd. 33 near intersection with Rd. 40, 28.VI.2000, F. Soto (1 specimen).

Additions to the original description.

**Head.** Dorsal chaetotaxy as in Fig. 57: macrochaetae A0, (A2), A3, S3, S5, Pa5 and Pm3 present. Postlabium with all setae ciliate; columns ICELO with 41232; posterior setae on column C detached from anterior group.

**Body.** Mesothorax as in *Trogolaphysa jataca* (Fig. 53). Metathorax as in (Fig. 58). Abd. 1 with 1 anterior (a6) and 4 posterior setae (Fig. 59). Abd. 2 and 3 as in *Trogolaphysa giordanoae* sp. n. (Figs 15, 16). Abd. 4 as in Fig. 60: inner macrochaetae A4, A5, B4, B5 and B6 present; outer macrochaetae D3, E2, E3, F1, F2, F3, Fe3 present; at least one other outer macrochaeta present. Posterior setae 17+17. Intersegmental membrane between Abd. 4–5 with 4–6 lenticular organs (Fig. 60).

**Ventral tube.** Anterior face with 4+4 distal macrochaetae.

**Remarks**: The individual from St. Thomas lacks head macrochaetae A2. In the individual from Aguadilla the dorsal and outer teeth of the unguis end on the basal fourth of the claw instead of the distal half.

## Discussion

### Dorsal chaetotaxy

The dorsal chaetotaxy of *Trogolaphysa* has not been fully described in the context of the AMS (Soto-Adames 2010) and Szeptycki (1979) systems of nomenclature. The notes presented below are based on the study of *Trogolaphysa jataca*, *Trogolaphysa geminata* and *Trogolaphysa riopedrensis*, three surface species from Puerto Rico, in addition to the three species of cave *Trogolaphysa* from Belize.

*Head*. The dorsal chaetotaxy of the head is reduced when compared to other genera of scaled Entomobryidae (e.g., *Seira*, *Pseudosinella*; cf. Fig. 26 here to fig. 1 in Soto-Adames [2008] and fig. 4 in Soto-Adames [2010]). In the species studied, series A includes setae A0-3. Some species have additional microchaetae that can be construed as belonging to this series (e.g., *Trogolaphysa jataca*, Fig. 52) but only A0–3 are present in all species examined. Seta A1 is always a normal, coarsely ciliate microchaeta, all other members of the series can develop into macrochaetae.

Series M includes 2 setae, probably homologous to M3–M4. In most species the lateral seta in series M is internal to S5, but in troglomorphs *Trogolaphysa jacobyi* sp. n. and *Trogolaphysa belizeana* the seta is inserted external to S5 and just internal to the dorsal cephalic suture. M0 is absent (seen only in one individual of *Trogolaphysa giordanoae* sp. n.), whereas M3 is often developed into a macrochaetae. Series S includes setae S1–5, S0 is absent (seen only in one individual of *Trogolaphysa geminata*). Among the species examined only setae S3 and S5 are modified into macrochaetae. Most setae in series S are inserted along the dorsal cephalic sulcus; the exceptions are S1, which is anterior to all others, and seta S3 when it is modified into a macrochaeta (cf., *Trogolaphysa giordanoae* sp. n. [Fig. 5] versus *Trogolaphysa jacobyi* sp. n. [Fig. 26]).

There is a pattern in the addition of macrochaetae on the interocular region of the head for species with 3–4 macrochaetae, but the pattern in not retained for species with five macrochaetae: whenever three macrochaetae are present they are always A0, A2 and M3; the species with four macrochaetae carries A0, A2 and M3 plus S3; the species with five macrochaetae have A0, A2, S3, S5, and either A3 or M3.

Series Ps includes only two setae (Ps2 and Ps5) whereas series Pa has four setae (Pa2, 3, 5 and bothriotrix Pa6), and series Pm and Pp has one seta each (Pm3 and Pp3). Posterior setae Pa5 and Pm3 are often modified into macrochaetae.

*Mesothorax*. The chaetotaxy of the mesothorax is reduced, as in scaled Entomobryidae (e.g., *Seira*, *Pseudosinella* [Soto-Adames 2008, 2010]), the closest group of Entomobryoidea for which detailed information about chaetotaxy is available. All *Trogolaphysa* species share the presence of macrochaetae a5 and p3, and microchaetae m2, m4, p4, p5, and what we provisionally call p6. Setae p1 and p2 are present in the three species from Belize but either absent or integrated in the p3 macrochaetae complex in the three surface species from Puerto Rico (Fig. 53)

The homologies of the posterior macrochaetae across the species examined are unclear. The presence of setae p1 and p2 in *Trogolaphysa giordanoae* sp. n. suggests that the cluster of six posterior macrochaetae represent a multiplication of seta p3; whereas the transformation of p1 and p2 into macrochaetae in *Trogolaphysa jacobyi* sp. n. and *Trogolaphysa belizeana*, and their absence in the surface species *Trogolaphysa jataca*, *Trogolaphysa geminata* and *Trogolaphysa riopedrensis* suggest that the three setae have been integrated (and duplicated) into the macrochaetal complex. We propose three hypotheses to explain the evolution of posterior macrochaeta: the macrochaetae evolved independently more than once in the genus, either as 1) a duplication of p1–3 or as 2) multiplication of p3 alone; 3) the cluster evolved only once, a duplication of p1–3, and the setae we have identified as p1 and p2 in *Trogolaphysa giordanoae* sp. n. are secondary and not homologous to those present in *Trogolaphysa jacobyi* sp. n. and *Trogolaphysa belizeana*. A study of the postembryonic development of these setae or molecular phylogenetic analysis may provide evidence in support one of the hypotheses proposed above.

*Metathorax*. The chaetotaxy of this segment is reduced to five setae (e.g., *Trogolaphysa geminata*, Fig. 58). The homologies of these setae are uncertain, and names provided in Fig. 58 are based on comparison with the general organization of the chaetotaxy in first instar *Seira dowlingi* (Wray, 1953), *Heteromurus nitidus* (Templeton, 1835) and *Willowsia buskii* (Lubbock, 1870) (Soto-Adames 2008, Szeptycki 1979). The single macrochaeta present in *Trogolaphysa jacobyi* sp. n. appears to be homologous to p3, whereas the three macrochaetae present in *Trogolaphysa giordanoae* sp. n. and *Trogolaphysa belizeana* appear to be homologous to a displaced a2, p2 and p3.

*Abdomen 1*. This segment also has a reduced chaetotaxy, carrying not more than six setae (Figs 14, 59). The homologies proposed are based on comparisons with first instar *Seira dowlingi*, *Heteromurus nitidus* and *Willowsia buskii* (Soto-Adames 2008, Szeptycki 1979). Seta a6 is present in *Trogolaphysa giordanoae* sp. n., *Trogolaphysa geminata* and *Trogolaphysa riopedrensis* and absent in *Trogolaphysa jacobyi* sp. n. and *Trogolaphysa jataca*.

*Abdomen 2*–*3.* The chaetotaxy of these segments was previously described by Mari Mutt (1987[1988]) and the species examined here, including *Trogolaphysa belizeana*, conform to that description. These two segments do not carry inner microchaetae beyond those associated with the bothriotichal complexes. The macrochaetae on Abd. 2 are homologous to m3 and m5. Lateral setae a6, m6 and p5 appear to be present in all species, although a6 and m6 are often difficult to see. The socket of p5 is enlarged, macrochaeta-like, but this seta falls off in most slide-mounted individuals, it was observed in *Trogolaphysa jacobyi* sp. n, where it is a ciliate mesochaeta and in *Trogolaphysa jataca*, where it is enlarged and fusiform (Fig. 54).

The macrochaetae on Abd. 3 appear to be homologous to m3, am6, pm6 and p6 (Fig. 16). Sensillum d2 is absent in *Trogolaphysa jacobyi* sp. n. (Fig. 37), in *Trogolaphysa belizeana* it is inserted posterior to macrochaeta pm6 (Fig. 48), whereas in *Trogolaphysa giordanoae* sp. n., *Trogolaphysa jataca*, *Trogolaphysa geminata* and *Trogolaphysa riopedrensis* it is inserted anterior to or forming a row with pm6 (Fig. 16).

*Abdomen 4.* The chaetotaxy of Abd. 4 is similar to that in scaled Entomobryidae and setae modified in, for example, *Seira* or *Lepidocyrtus*, can also be modified in *Trogolaphysa*. The chaetotaxy displays some unique peculiarities. For example, what appears to be seta B6 is, in most species, a meso- or small macrochaeta inserted just posterior to B5 (Fig. 17). In addition, the posterior bothriotrix corresponds to D4 (D3 in *Seira*, Soto-Adames 2008). The number, identity and relative insertion of inner macrochaetae varies between *Trogolaphysa* species. *Trogolaphysa giordanoae* sp. n. and *Trogolaphysa riopedrensis* share the same inner macrochaetae (A4, A5, B4, B5), but the insertion of B4 in relation to the pseudopore and seta C2 differ between these two species (cf., Figs 17, 60). *Trogolaphysa geminata*, *Trogolaphysa jataca* and *Trogolaphysa belizeana* have three inner macrochaetae and share macrochaetae A5 and B5, but whereas in *Trogolaphysa jacobyi* sp. n. the third macrochaetae is B4, in the other two species it is A4. *Trogolaphysa jacobyi* sp. n. is also unusual in having macrochaeta B5 displaced towards A6 instead of C4 (Fig. 38).

The external macrochaetae in the first three rows of columns D, E and F are stable in the species of examined. All species have macrochaetae D3, E2, E3, F1 and F2. Macrochaeta F3 is present in all species except *Trogolaphysa jacobyi* sp. n. The number of macrochaetae external to column F and posterior to row 3 varies intra- and interspecifically. However, the lateral and posterior fields are often difficult to see in regular preparations and it is possible that some of the apparent differences are simply incomplete observations.

The number of posterior setae (per side) on Abd. 4 also varies between species: 6–7 in *Trogolaphysa belizeana* and *Trogolaphysa jacobyi* sp. n., 13–14 in *Trogolaphysa jataca* and *Trogolaphysa geminata*, 17 in *Trogolaphysa riopedrensis* and 19–21 in *Trogolaphysa giordanoae* sp. n.

### Chaetotaxy and phylogenetic analysis of cave-adapted species

The morphological information for surface species *Trogolaphysa luquillensis* (Mari Mutt 1987[1988]), cave species *Trogolaphysa subterranea* (Mari Mutt 1987[1988]) and the six species treated here was coded into 69 characters (Appendix 1). The data matrix (Appendix 2) includes character systems identified (Christiansen 1961, 1965; Gama 1984) as most responsive to adaptation to cave habitats (i.e., eye number, claw complex morphology), but most characters (60) refer to chaetotaxy. *Campylothorax sabanus* (Wray, 1953) was designated as outgroup.

**Figure 61–64.**
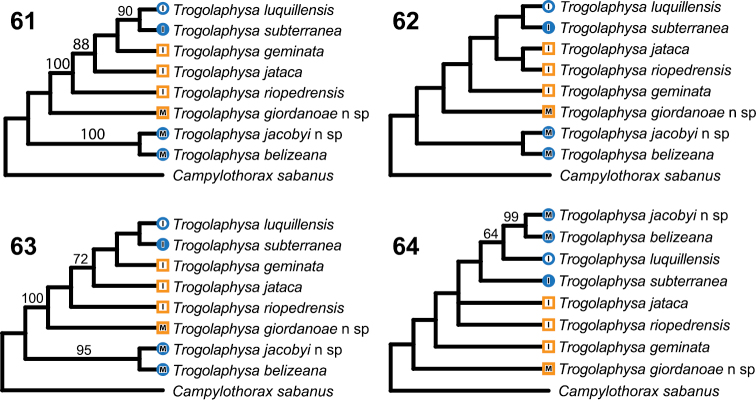
Cladograms. Branch lengths are arbitrary. All searches performed using branch and bound, including the bootstrap analyses. Numbers above branches are bootstrap values based on 5000 pseudoreplicates. Circles: taxa with troglomorphies, squares-not troglomorphic. Solid symbols recorded only from caves, open symbols recorded from surface. M, mainland species: I, island species **61**–**62** The two shortest trees found when all characters are included in the analysis **63** Shortest tree found when only chaetotactic characters are analyzed **64** Shortest tree found when only eye number, characters related to claw complex morphology and mucro are analyzed.

Phylogenetic analysis based on all characters supports two equally parsimonious trees (Figs 61–64) in which the two troglobiontic species from Belize form a monophyletic group and *Trogolaphysa giordanoae* sp. n. is placed at the base of the species from Puerto Rico. The parsimony trees support the sister species relationship between *Trogolaphysa subterranea* and *Trogolaphysa luquillensis*, but relationships between the other three species from Puerto Rico are unresolved, as *Trogolaphysa riopedrensis* is placed as sister to either *Trogolaphysa jataca* or to a clade that includes all other island species.

The apparently rare occurrence of metathoracic macrochaetae in the three Belizean species suggests a close relationship between them, but the parsimony trees show the troglobiontic species diverging before the separation of *Trogolaphysa giordanoae* sp. n. from the ancestor of the island species. The lack of support for the monophyly of Belizean species may be an artifact of a disproportionate contribution of characters under strong cave habitat selection to the final topology of the tree. However, parsimony analysis based only on chatotactic characters results in a single shortest tree (Fig. 63), which also supports the monophyly of troglobiontic species while retaining *Trogolaphysa giordanoae* sp. n. at the base of the island species clade.

To assess whether putative adaptive characters provide support for alternative relationships, we conducted a phylogenetic analysis using only eye number, ornamentation of labral papilla, and claw and mucro morphology. These characters support a single tree (Fig. 64) that places most surface forms at the base of the tree while supporting a clade comprising the cave species (*Trogolaphysa jacobyi* sp. n., *Trogolaphysa belizeana*, *Trogolaphysa subterranea*) and *Trogolaphysa luquillensis*. *Trogolaphysa luquillensis* is endemic to the tropical rainforest and is unique among surface species examined here in having an acuminate tenent hair and three inner ungual teeth close to each other and inserted in the basal half of the claw. These characters of the claw have been identified as adaptations to walking on water surface or other, permanently wet, surfaces such as those found in rainforest leaf litter and caves (Christiansen 1961, 1965).

Evaluation of the direction of evolution of head chaetotaxy using trees in Fig. 61 and Fig. 62 supports a trend towards a reduction in number of macrochaetae. However, the pattern is equivocal because some macrochaetae may be lost independently through out the tree, depending on tree topology. For example, S5 might have been lost once and regained or it might have been lost twice independently. What is clear from this analysis is that A3 is the first macrochaetae to be lost, followed by S5, S3 and M3 (Table 4). *Trogolaphysa riopedrensis* is the only species in which this pattern seems to be disrupted: under either tree this species is hypothesized to have lost M3 and gain A3 independently.

**Table 4.** Distribution of head macrochaetae in eight species of New World *Trogolaphsya*.

**Species**	**Macrochaetae number**	**Macrochaeta identity**					
*Trogolaphysa jacobyi* sp. n.	6	A0	A2	A3	M3	S3	S5
*Trogolaphysa belizeana*	6	A0	A2	A3	M3	S3	S5
*Trogolaphysa riopedrensis*	5	A0	A2	A3	—	S3	S5
*Trogolaphysa jataca*	5	A0	A2	—	M3	S3	S5
*Trogolaphysa geminata*	4	A0	A2	—	M3	S3	—
*Trogolaphysa luquillensis*,	3	A0	A2	—	M3	—	—
*Trogolaphysa giordanoae* sp. n.	3	A0	A2	—	M3	—	—
*Trogolaphysa subterranea*	2	A0	A2	—	—	—	—

### Taxonomic status of *Dicranocentruga* and *Trogolaphysa*

The character used by Mitra (1993, 2002) to diagnose genera *Trogolaphysa* and *Dicranocentruga* can be difficult to apply. The presence of EOS is difficult to ascertain using phase contrast or DIC light microscopy. The retention of *Dicranocentruga* as a valid genus hinges on whether *Trogolaphysa maya*, the type species of *Trogolaphysa*, carries the EOS. As pointed out above, the presence of EOS in the two troglomorphic species considered here suggests that this structure is also present in *Trogolaphysa maya*. We examined the single alcohol preserved syntype of *Trogolaphysa maya* deposited at the Illinois Natural History, but the condition of the specimen is such that confirmation of the presence of the EOS is impractical.

It is possible, as proposed by Mitra (1993), that a more extensive analysis of idiochaetotaxy may provide diagnostic characters for these two genera that are easier to see and interpret. The present study does not support this idea. The organization of the idiochaetotaxy is the same in all the species studied. Changes in the distribution of setae, as in the case of the metathorax, are related to the morphology of the elements (whether macro- or microsetae), and not to the presence of EOS, number of eyes, or other cave adaptive characters. Until such time as the presence of EOS can be reliably determined, or other diagnostic characters are found, we retain all New World *Dicranocentruga* in the genus *Trogolaphysa*, as proposed by Thibaud and Najt (1988[1989]).

### Morphological characters and phylogeny

**Figure 65.**
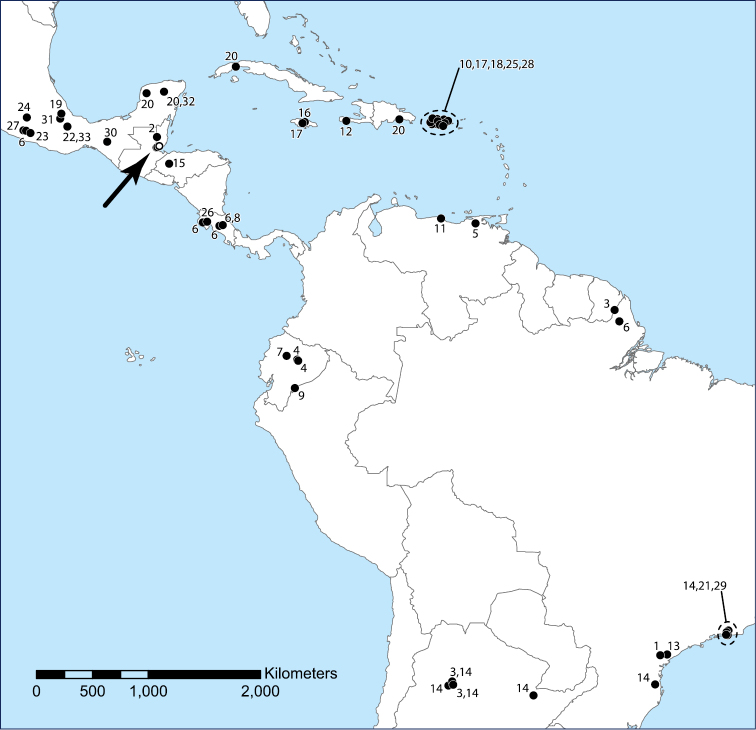
Central and South America and the Caribbean Islands, showing the published distributions of described New World species of the genus *Trogolaphysa*. Open circles (arrow): *Trogolaphysa jacobyi* sp. n., *Trogolaphysa giordanoae* sp. n. Closed circles: **1** *Trogolaphysa aelleni* Yoshii, 1988 **2** *Trogolaphysa belizeana* **3** *Trogolaphysa berlandi* (Denis, 1925) **4** *Trogolaphysa bessoni* Thibaud & Najt, 1989 **5** *Trogolaphysa caripensis* (Gruia, 1987) **6** *Trogolaphysa carpenteri* (Denis, 1925) **7** *Trogolaphysa cotopaxiana* Thibaud & Najt, 1989 **8** *Trogolaphysa distinguenda* (Denis, 1931) **9** *Trogolaphysa ecuatorica* (Palacios-Vargas, Ojeda & Christiansen, 1986) **10** *Trogolaphysa geminata* **11** *Trogolaphysa guacharo* Yoshii, 1988 **12** *Trogolaphysa haitica* (Palacios-Vargas, Ojeda & Christiansen, 1986) **13** *Trogolaphysa hauseri* Yoshii, 1988 **14** *Trogolaphysa hirtipes* (Handschin, 1924) **15** *Trogolaphysa hondurasensis* (Palacios-Vargas, Ojeda & Christiansen, 1986) **16** *Trogolaphysa jamaicana* (Palacios-Vargas, Ojeda & Christiansen, 1986) **17** *Trogolaphysa jataca* **18** *Trogolaphysa luquillensis* **19** *Trogolaphysa marimutti* (Palacios-Vargas, Ojeda & Christiansen, 1986) **20** *Trogolaphysa maya* **21** *Trogolaphysa millsi* Arlé, 1939 **22** *Trogolaphysa nacionalica* (Palacios-Vargas, Ojeda & Christiansen, 1986) **23** *Trogolaphysa oztotlica* (Ojeda & Palacios-Vargas, 1984) **24** *Trogolaphysa relicta* (Palacios-Vargas, Ojeda & Christiansen, 1986) **25** *Trogolaphysa riopedrensis* **26** *Trogolaphysa separata* (Denis, 1933) **27** *Trogolaphysa strinatii* Yoshii, 1988 **28** *Trogolaphysa subterranea* **29** *Trogolaphysa tijucana* (Arlé & Guimarāes, 1979) **30** *Trogolaphysa toroi* (Palacios-Vargas, Ojeda & Christiansen, 1986) **31** *Trogolaphysa variabilis* (Palacios-Vargas, Ojeda & Christiansen, 1986) **32** *Trogolaphysa xtolokensis* (Palacios-Vargas, Ojeda & Christiansen, 1986) **33** *Trogolaphysa yoshiia* (Palacios-Vargas, Ojeda & Christiansen, 1986).

The genus *Trogolaphysa* has diversified in the New World from where now 35 species have been named (Table 1, Fig. 65), many of which are troglobionts or at least eutroglophiles (*sensu* Sket 2008, Culver and Pipan 2009). Phylogenetic studies of species-level relationships have not been published for this genus, perhaps as a result of the scarcity and quality of the characters available for analysis. Most described species, especially cave forms, have been diagnosed almost exclusively based on characters of the claw complex, mucronal shape and development of the antennae, characters identified as malleable under selective pressures (Christiansen 1961). A new set of characters or character systems, would be needed to perform more reliable phylogenetic analyses. Most other studies addressing the evolution of morphological convergence in cave-adapted arthropods have used molecular data (e.g., Trontelj et al. 2012) to generate phylogenies for hypothesis testing. However, many troglobiontic springtail species are known only from a few individuals from few, seldom visited localities (as is evident from the small number of records reported in Mari Mutt and Bellinger 1990, 1996, and Mari Mutt et al. 2009), which are not suitable or available for molecular analysis. For these species only morphological information can be used to evaluate the evolution of other morphological characters.

Ever since the publication of Gisin’s (1967) “systématique ideal,” collembolan systematists have assumed that ideochaetotaxic characters are non-adaptive characters that evolve neutrally, are less prone to convergence and, therefore, more valuable for phylogenetic analysis. However, this assumption has never been tested in a phylogenetic context. The simple test performed here supports the traditional view of chaetotaxy as less vulnerable to directional convergence than characters related to claw structure. Analysis based exclusively on putative cave-adaptive characters support a clade comprising cave species from Puerto Rico and Belize, whereas analysis of chaetotaxy alone supports the placement of cave species from Puerto Rico and Belize in independent clades. Despite the clear difference in signal in the character partitions it should be noted that analysis of the complete character set results in higher bootstrap values for what is basically the chaetotaxy-only tree, than when only chaetotactic characters are analyzed. It is clear that some putative adaptive characters retain phylogenetic information concordant with chaetotaxy characters, an observation which argues in favor of the retention of all characters in the analysis. The simple test preformed here has to be expanded to include many more species, to determine if the result obtained are consistent or just an artifact of the sparse taxon sampling. It is unclear if chaetotaxy will provide sufficient characters to resolve relationships in an analysis that includes all species. In any case, there are problems related to the evolution and homology of some chaetotactic characters (e.g., posterior macrochaetae on the meso- and metathorax, and the inner macrochaetae on the fourth abdominal segment) that may be intractable on morphology-based datasets, and will require the use of putatively independent molecular characters.

### Habitats

**Figure 66–69.**
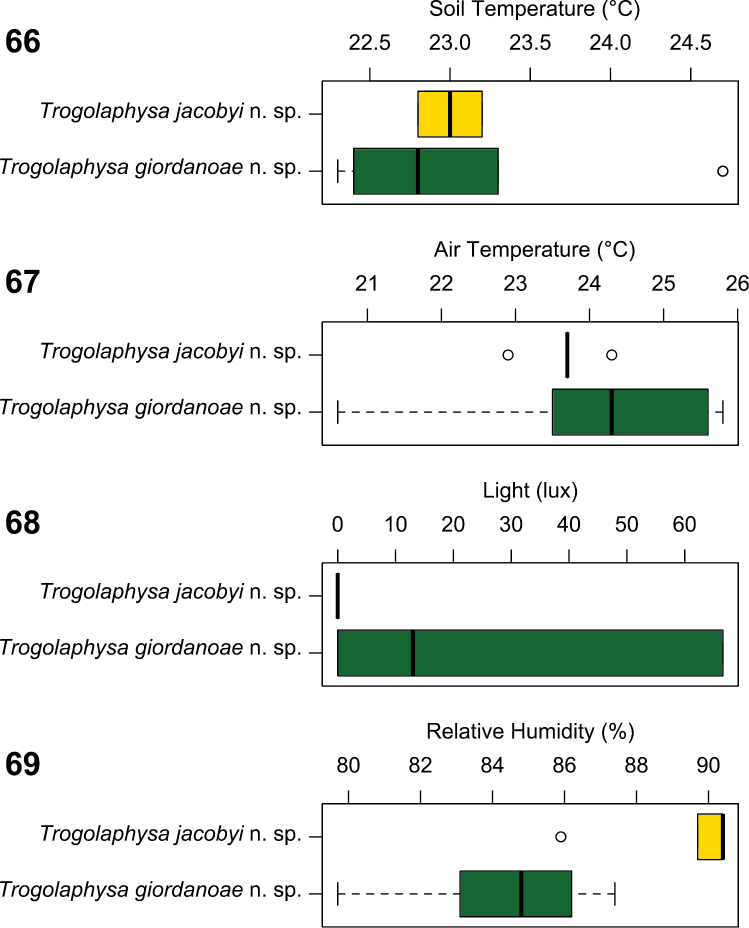
Boxplot comparisons of environmental parameters for collections of *Trogolaphysa jacobyi* sp. n. and *Trogolaphysa giordanoae* sp. n. **66** Soil temperature **67** Air temperature **68** Light **69** Relative humidity.

The two new species were found in conditions of similar substrate (*Trogolaphysa jacobyi* sp. n. mean=23.0 °C; *Trogolaphysa giordanoae* sp. n. mean=23.1 °C; W=11, p=0.7200) (Fig. 66) and air temperatures (*Trogolaphysa jacobyi* sp. n. mean=23.7 °C; *Trogolaphysa giordanoae* sp. n. mean=24.3 °C; W=23.5, p=0.3947) (Fig. 67), but *Trogolaphysa jacobyi* sp. n. was found only in complete darkness (Fig. 68), whereas *Trogolaphysa giordanoae* sp. n. was found at significantly brighter and varying light conditions, typically in twilight (*Trogolaphysa jacobyi* sp. n. mean=0.0 lux; *Trogolaphysa giordanoae* sp. n. mean=29.5 lux; W=12.5, p=0.0260). *Trogolaphysa jacobyi* sp. n. also was found primarily under conditions of significantly elevated humidity, whereas *Trogolaphysa giordanoae* sp. n. was more varied in the humidity levels at which it was found (*Trogolaphysa jacobyi* sp. n. mean=89.36 %; *Trogolaphysa giordanoae* sp. n. mean=84.56 %; W=65, p=0.0056) (Fig. 69). In addition, *Trogolaphysa giordanoae* sp. n. was frequently associated with fruit bat guano or other scat (Fig. 23). These observations support our classification of *Trogolaphysa jacobyi* sp. n. as a troglobiont and *Trogolaphysa giordanoae* sp. n. as a guanophile.

## Supplementary Material

XML Treatment for Trogolaphysa

XML Treatment for Trogolaphysa giordanoae

XML Treatment for Trogolaphysa jacobyi

XML Treatment for Trogolaphysa belizeana

XML Treatment for Trogolaphysa jataca

XML Treatment for Trogolaphysa geminata

XML Treatment for Trogolaphysa riopedrensis

## Appendix I

Character and character states as circumscribed for phylogenetic analysis. (doi: 10.3897/zookeys.323.4950.app1) File format: Microsoft Word document (doc).

## Appendix II

Data matrix of morphological characters used in the phylogenetic analysis. (doi: 10.3897/zookeys.323.4950.app2) File format: Microsoft Word document (doc).
