# Supplementary material for: The dorsal chaetotaxy of Trogolaphysa (Collembola, Paronellidae), with descriptions of two new species from caves in Belize
Source: Zookeys. 2013 Aug 13;(323):35–74. doi: 10.3897/zookeys.323.4950 (PMC3760292; doi:10.3897/zookeys.323.4950)
Supplement: Supplementary file 8 — Character and character states as circumscribed for phylogenetic analysis. (doi: 10.3897/zookeys.323.4950.app1) File format: Microsoft Word document (doc). [file ZooKeys-323-035-s001.doc]

Appendix 1. Character and character states as circumscribed for phylogenetic analysis.

1. Eye Number: The number of eyes is often difficult to ascertain. Species with 6 eyes are said to have 8+8, with eyes G and H reduced, whereas *T. jacobyi* sp. n. has pigment in the eyepatch visible in living individuals but no eyes were visible under regular phase compound microscope. Species were scored according to what was observed under a phase compound microscope.

0: absent 5: five

1: one 6: six

2: two 7: seven

3: three 8: eight

4: four

Head setae nomenclature follows the AMS systems (Soto-Adames 2010) as in Fig. 26.

2. Head Seta A0

0: absent

1: microseta

2: Macroseta

3. Head Seta A2

0: absent

1: microseta

2: Macroseta

4. Head Seta A3

0: absent

1: microseta

2: Macroseta

5. Head Seta A4

0: absent

1: microseta

2: Macroseta

6. Head Seta M3

0: absent

1: microseta

2: Macroseta

7. Head Seta M4

0: absent

1: microseta

2: Macroseta

8. Head Seta S1

0: absent

1: microseta

2: Macroseta

9. Head Seta S2

0: absent

1: microseta

2: Macroseta

10. Head Seta S3

0: absent

1: microseta

2: Macroseta

11. Head Seta S4

0: absent

1: microseta

2: Macroseta

12. Head Seta S5

0: absent

1: microseta

2: Macroseta

13. Head Seta Pa5

0: absent

1: microseta

2: Macroseta

14. Head Seta Pm3

0: absent

1: microseta

2: Macroseta

Labral rows nomenclature follows Palacios Vargas et al. (1985[1986])

15. pre-Labral Setae

0: smooth

1: ciliate

16. Labrum Row A

0: all subequal

1: A0 shortest

2: A1 shortest

3: A2 shortest

17. Labrum Row B

0: all subequal

1: B0 shortest

2: B1 shortest

3: B2 shortest

18. Labrum Row C

0: all subequal

1: C0 shortest

2: C1 shortest

19. Labrum Distal Margin

0: smooth

1: denticulate

20. Outer Maxillary Lobe Apical Seta

0: smooth

1: ciliate

21. Outer Maxillary Lobe Sublobal Plate Appendages

0: none

1: one

2: two

3: three

4: four

22. Labium M1

0: absent

1: smooth

2: ciliate

23. Labium M2

0: absent

1: smooth

2: ciliate

24. Labium r

0: absent

1: reduced smooth

2: reduced ciliate

3: normal smooth

4: normal ciliate

25. Labium E

0: absent

1: smooth

2: ciliate

26. Labium L1

0: absent

1: smooth

2: ciliate

27. Labium L2

0: absent

1: smooth normal

2: ciliate normal

3: smooth reduced

28. post-Labium Scales

0: absent

1: present

29. post-Labium Ornamentation Anterior Row Setae

0: smooth

1: ciliate

30. post-Labium Column I Setae Number

0: none 5: five

1: one 6: six

2: two 7: seven

3: three 8: eight

4: four 9: nine or more

31. Post-Labium Column I Detached Seta: This character refers to the relative distance between setae on the column. The most posterior seta is often separated from the penultimate seta by a distance larger than that between the anterior setae (Fig. 11)

0: none

1: one

2: two

32. Mesothorax Macrosetae Anterior

0: absent 5: five

1: one 6: six

2: two 7: seven

3: three 8: eight

4: four 9: nine or more

Circumscription of characters and character states for the inner posterior chaetotaxy of the mesothorax is problematic. As explained in the Dorsal Chaetotaxy section, there are several possible hypotheses for the homology of p1, p2, p3 and the macrochaetae, and a particular character coding can reflect only a few such hypotheses. The character coding provided below considers the group of 6 posterior macrochaetae to be a multiplet of seta p3. See main text for additional comments.

33. Mesothorax p1

0: absent

1: microseta

2: Macroseta

34. Mesothorax p2

0: absent

1: microseta

2: Macroseta

35. Mesothorax p3

0: absent

1: microseta

2: 1 Macroseta

3: 2 Macrosetae

4: 3 Macrosetae

5: 4 Macrosetae

6: 5 Macrosetae

7: 6 Macrosetae

36. Metathorax a2

0: absent

1: microseta

2: Macroseta

37. Metathorax p2

0: absent

1: microseta

2: Macroseta

38. Metathorax p3

0: absent

1: microseta

2: Macroseta

39. Metathorax m4

0: absent

1: microseta

2: Macroseta

40. First Abdominal Segment Microseta a6

0: absent

1: present

41. First Abdominal Segment Posterior Microseta

0: absent 5: five

1: one 6: six

2: two 7: seven

3: three 8: eight

4: four 9: nine or more

42. Third Abdominal Segment d2

0: absent

1: near am6

2: posterior pm6

43. Fourth Abdominal Segment Seta A4

0: absent

1: microseta

2: Macroseta

44. Fourth Abdominal Segment Seta A5

0: absent

1: microseta

2: Macroseta

45. Fourth Abdominal Segment Seta A6

0: absent

1: microseta

2: Macroseta

46. Fourth Abdominal Segment Seta Seta B4

0: absent

1: microseta

2: Macroseta

47. Fourth Abdominal Segment Seta B4 Position

0: absent

1: anterior pseudopore

2: posterior pseudopore

48. Fourth Abdominal Segment Seta B5

0: absent

1: microseta

2: Macroseta

49. A4. Seta B5 Position

0: absent

1: near B6

2: near A6

50. Fourth Abdominal Segment Seta B6

0: absent

1: microseta

2: Macroseta

51. Fourth Abdominal Segment Seta C2 Position

0: absent

1: anterior paired T5

2: posterior T5

52. Fourth Abdominal Segment Seta C4 Position

0: absent

1: anterior paired B6

2: posterior B6

53. Fourth Abdominal Segment Seta D2 Position

0: absent

1: anterior or paired T3

2: posterior T3

54. Fourth Abdominal Segment Seta D3

0: absent

1: microseta

2: Macroseta

55. Fourth Abdominal Segment Seta E1

0: absent

1: microseta

2: Macroseta

56. Fourth Abdominal Segment Seta E2

0: absent

1: microseta

2: Macroseta

57. Fourth Abdominal Segment Seta E3

0: absent

1: microseta

2: Macroseta

58. Fourth Abdominal Segment Seta F1

0: absent

1: microseta

2: Macroseta

59. Fourth Abdominal Segment Seta F2

0: absent

1: microseta

2: Macroseta

60. Fourth Abdominal Segment Seta F3

0: absent

1: microseta

2: Macroseta

61. Fourth Abdominal Segment Posterior Setae

0: absent 5: five

1: one 6: six

2: two 7: seven

3: three 8: eight

4: four 9: nine or more

62. Hind Legs Tenent Hair

0: acuminate

1: spatulate

63. Hind Legs Claw Teeth

0: absent

1: one

2: two

3: three

4: four

64. Hind Legs Position Basal Teeth

0: absent

1: basal fourth

2: basal half

3: distal half

4: distal fourth

65. Hind Legs Size of Basal Teeth

0: absent

1: subequal

2: one slightly larger

3: one much larger

66. Hind Legs Position Basal Unpaired Tooth

0: absent

1: basal fourth

2: basal half

3: distal half

4: distal fourth

67. Hind Legs Position Outer Tooth

0: absent

1: basal fourth

2: basal half

3: distal half

4: distal fourth

68. Dens Rows Dental Spines

0: absent

1: one

2: two

69. Number Mucronal Teeth

0: absent

1: one

2: two

3: three

4: four

5: five
