# Supplementary material for: The dorsal chaetotaxy of Trogolaphysa (Collembola, Paronellidae), with descriptions of two new species from caves in Belize
Source: Zookeys. 2013 Aug 13;(323):35–74. doi: 10.3897/zookeys.323.4950 (PMC3760292; doi:10.3897/zookeys.323.4950)
Supplement: Supplementary file 9 — Data matrix of morphological characters used in the phylogenetic analysis. (doi: 10.3897/zookeys.323.4950.app2) File format: Microsoft Word document (doc). [file ZooKeys-323-035-s002.doc]

Appendix 2. Data matrix of morphological characters used in the phylogenetic analysis.

1.......10........20........30........40........50........60.......69

Trogolaphysa luquillensis 622102111111221030102221222114110070001?41220122121122122222903222124

Trogolaphysa subterranea 622101111111221030102221222114110070001?41220122121122122222903223124

Trogolaphysa geminata 622102111211221030102221222114110070001141220122121122122222914213124

Trogolaphysa jataca 622102111212221030102221222114110070001041220122121122122222914223124

Trogolaphysa riopedrensis 622201111212221030102221222114110070001141220212121222122222914223324

Trogolaphysa giordanoae sp n 622102111111221030102222222114111172221141220222121122122222914213124

Trogolaphysa jacobyi sp n 022202111212220030000211111019012221121040120222222212122222703112123

Trogolaphysa belizeana 022202111212220???000211111017012222221042220122121112122222702110023

Campylothorax sabanus 822220002222201210002223113115202262222042222222122112022222914213224
